# Multifunctional nanomedicines-enabled chemodynamic-synergized multimodal tumor therapy via Fenton and Fenton-like reactions

**DOI:** 10.7150/thno.80887

**Published:** 2023-03-21

**Authors:** Haiyan Gao, Zhiping Cao, Huanhuan Liu, Lijuan Chen, Yan Bai, Qingxia Wu, Xuan Yu, Wei Wei, Meiyun Wang

**Affiliations:** 1 Department of Medical Imaging, Henan Provincial People's Hospital & the People's Hospital of Zhengzhou University, Zhengzhou, 450003, P. R. China.; 2 School of Biomedical Sciences, Faculty of Medicine, The Chinese University of Hong Kong, Shatin, NT, Hong Kong SAR, 999077, China.; 3 Laboratory of Brain Science and Brain-Like Intelligence Technology, Institute for Integrated Medical Science and Engineering, Henan Academy of Sciences, Zhengzhou, 450003, P. R. China.

**Keywords:** Chemodynamic therapy, Combination therapies, Tumor microenvironment, Reactive oxygen species, Nanotechnology

## Abstract

Chemodynamic therapy (CDT) is well-known for using the tumor microenvironment to activate the Fenton reaction or Fenton-like reaction to generate strong oxidative hydroxyl radicals for tumor-specific treatment. It is highly selective and safe, without depth limitation of tissue penetration, and shows its potential as a new green therapeutic method with great clinical application. However, the catalytic efficiency of reagents involved in the Fenton reaction is severely affected by the inherent microenvironmental limitations of tumors and the strict Fenton reaction-dependent conditions. With the increasing application of nanotechnology in the medical field, combined therapies based on different types of functional nanomaterials have opened up new avenues for the development of next-generation CDT-enhanced system. This review will comprehensively exemplify representative results of combined therapies of CDT with other antitumor therapies such as chemotherapy, phototherapy, sonodynamic therapy, radiation therapy, magnetic hyperthermia therapy, immunotherapy, starvation therapy, gas therapy, gene therapy, oncosis therapy, or a combination thereof for improving antitumor efficiency from hundreds of the latest literature, introduce strategies such as the ingenious design of nanomedicines and tumor microenvironment regulations to enhance the combination therapy, and further summarize the challenges and future perspective of CDT-based multimodal anticancer therapy.

## Introduction

Cancer is the second leading cause of death worldwide and brings huge economic burden on families and societies 1. According to the latest global cancer burden figures for 2020 released by the World Health Organization's International Agency for Research on Cancer (IARC), 19.29 million new cancer cases and 9.96 million cancer deaths occurred worldwide in 2020. Globally, the cancer burden is expected to increase by 50% in 2040 compared to 2020 due to the aging population that is supposed to lead to nearly 30 million new cancer cases. The number of malignant tumor incidences and deaths in China continues to rise, and the annual medical expenditure due to malignant tumors exceeds $220 billion 2. Thus, exploring efficient treatments and developing innovative anti-cancer drugs will be highly beneficial for reducing the cancer burden and prolonging patients' life expectancy.

Reactive oxygen species (ROS), mainly including superoxide anion (•O_2_^-^), singlet oxygen (^1^O_2_), hydroxyl radicals (•OH), and hydrogen peroxide (H_2_O_2_), are a class of toxic substances that damage cells 3-5. ROS can damage biological macromolecules such as lipids, proteins, and DNA, and thereby induce cancer cell apoptosis 6-8. In 2019, Shi's group proposed the concept of “reactive oxygen species science” as an emerging scientific discipline, which systematically expounds on the chemical mechanism, biological effects, and therapy applications of ROS, providing feasible ROS-related cancer treatment strategies 9. Meanwhile, the rapid development of nanotechnology in recent years has achieved many gratifying results in cross-application with medicine, especially in the field of nanodrug carriers, combination therapies and targeted therapies, which lead to new directions in cancer treatment 10-12. To date, researchers have prepared a variety of nanomaterials with unique ROS regulation properties, which are widely used in the biomedical field 13-15. Many anti-tumor approaches can induce ROS production, for instance, chemotherapy, photodynamic therapy (PDT), radiation therapy (RT), sonodynamic therapy (SDT), and chemodynamic therapy (CDT). Compared with other therapies, CDT has the advantages of high selectivity, no depth limitation of tissue penetration, evitable damage to normal tissues, and no need for exogenous energy in reaction activation.

CDT is a new type of tumor treatment technique based on the transformation reaction of endogenous chemical products in tumors using the Fenton or Fenton-like reactions. It has attracted widespread attention from the international academic community after it was proposed in 2016 16. Essentially, the Fenton reaction represents a process of Fe^2+^ catalyzing the highly expressed H_2_O_2_ in the tumor microenvironment (TME) to produce highly oxidized •OH (typical reaction scheme: Fe^2+^+H_2_O_2_→Fe^3+^+OH•+OHˉ; Fe^3+^+ H_2_O_2_→Fe^2+^+HO_2_•+H^+^). Many types of iron-based catalysts have been developed for the Fenton reaction, but their efficiency varies: only reagents that readily release Fe (II) exhibit good CDT performance. New century researchers have also explored Fenton-like reactions mediated by copper (Cu), manganese (Mn), cobalt (Co), molybdenum (Mo), titanium (Ti), tungsten (W) and zinc (Zn), vanadium (V), palladium (Pd), silver (Ag), cerium (Ce), ruthenium (Ru) for enhanced CDT efficacy 17-20. However, developing a Fenton catalyst with excellent CDT performance remains challenge. For example, it was reported that many manganese-based catalysts have been designed for Fenton-like reactions since MnO_2_ is easily reduced to Mn^2+^ by GSH in tumors 21. It is worth noting that Mn^2+^ exerts Fenton-like activity in the presence of HCO_3_ˉ and it is necessary to consider that the low concentration of HCO_3_ˉ in tumors will affect the performance of CDT reagents. Additionally, the CDT also still has the following limitations: (1) the pH (pH 6.5-6.9) in the TME is much higher than that required for the Fenton reaction (pH 2-4); (2) the content of H_2_O_2_ (100 μM-1 mM) in the tumor is not enough to produce sufficient •OH, while the overexpression of glutathione (GSH) in the TME (up to 10 mM) can consume •OH and reduce the treatment efficiency; (3) Low catalytic efficiency of Fenton reagents.

Focusing on the problems as mentioned above, several strategies have been developed to enhance the therapeutic efficacy of CDT, some of which are listed in **Table** 1. For example, modulating the TME states, such as lowing the pH of the tumor 22, designing Fenton reaction reagents that are less dependent on pH 23, 24, increasing the level of H_2_O_2_ 17, 25, 26, or consuming GSH in the tumor 27, 28, can improve the therapeutic outcome of CDT. Besides, optimizing the morphology and interface properties of the catalysts could enhance their catalytic ability, since the chemical composition, size, and aggregation of the nanocatalysts have a great impact on the catalytic rate and activity of the Fenton reaction 29. Nowadays, a variety of nanocatalysts (transition metal nanomaterials, metal-organic nanomaterials, monoatomic nanomaterials, and electron-rich nanomaterials) are synthesized to improve CDT efficiency 30-33. Moreover, adding physical energy fields such as light, temperature, ultrasonic field, X-ray, and magnetic field can also promote the speed of the Fenton reaction 34-39. Another option for CDT enhancement is modulating the nutrition or immune system of the tumor to reinforce the overall therapeutic effect 40. Numerous systems had been designed and developed based on these design principles to accelerate the Fenton reaction. And there are several excellent reviews about the enhanced CDT have been reported. For example, Prof. Bu has systematically described enhanced chemodynamic therapy strategies from the perspectives of material design, microenvironmental regulation of tumor area, and exogenous energy field regulation, respectively 41. Chen et al. also introduced intensive CDT strategies and CDT-based multimodal anti-cancer therapies 42. Although the CDT has also been reported in other biomedical applications, CDT-based combination therapies are primarily used in cancer treatment. However, a comprehensive review of recent advances in combining CDT with all other treatment modalities to improve cancer treatment outcomes, as well as the intelligent design of different nanomaterials to enable synergistic enhancement therapy, have not been systematically reported.

**Scheme 1:**
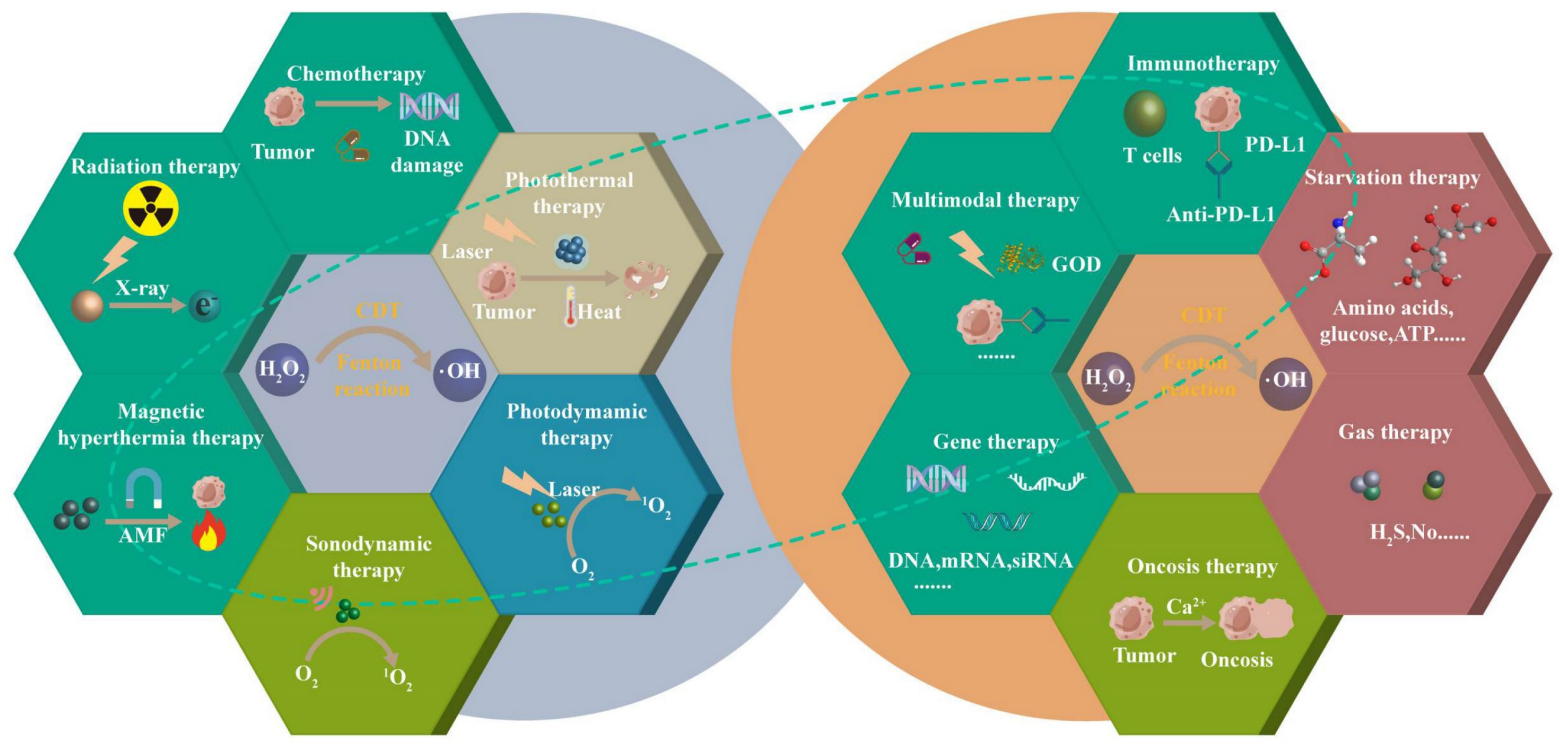
CDT combines different therapeutic approaches to achieve multimodal treatment strategies, including chemotherapy, photodynamic therapy, photothermal therapy, sonodynamic therapy, radiation therapy, magnetic hyperthermia therapy, immunotherapy, starvation therapy, gas therapy, gene therapy, oncosis therapy, or a combination thereof.

The purpose of this article is to review the latest progress of CDT combined with chemotherapy, phototherapy, SDT, RT, magnetic hyperthermia therapy (MHT), immunotherapy, starvation therapy (ST), gas therapy (GS), gene therapy (GT), oncosis therapy (OT), or a combination thereof (Scheme 1). CDT combined with ion interference therapy (IIT) and electrodynamic therapy (EDT) are also introduced. Each section mainly contains some representative rational designs and advances in CDT strategies, a summary of each treatment method and its future perspective, and the comparison of the advantages and disadvantages of different combination therapies in the ending. We want to provide readers with a more comprehensive understanding of each combination therapy's advantages, and the “1+1> 2” or “1+1+1>3” anti-tumor therapeutic effects. Finally, we also propose the challenges of CDT therapy and look forward to future development, especially the application prospects of microfluidic technology and cell membrane encapsulation technology in CDT-based combination therapy.

## CDT-based Combination therapy

The single treatment of CDT cannot completely eradicate the tumor, which paves the way for the combined treatment to enhance CDT. According to previous reports, CDT-based combination cancer treatments are being widely reported to achieve satisfactory treatment outcomes. We will show the representative examples of CDT/chemotherapy, CDT in combination with therapies based on external stimuli (light, ultrasound, x-rays, magnet) and chemical and biological stimuli (immunization, nutrition, gas, gene silencing, bioactive ions) for synergistic enhancement (**Table 2**).

### CDT in Combination with Chemotherapy

Chemotherapy, which uses highly toxic drugs to induce DNA bond breakage and interfere with DNA synthesis to kill cancer cells, has played a key role in cancer treatment 43. However, unsatisfactory drug delivery, suboptimal anti-tumor effects, severe side effects, and drug resistance have severely weakened its clinical outcomes. Numerous studies have shown that nanomedicines combining chemotherapy with CDT provide synergistic effects in inhibiting tumor growth and suppressing tumor metastasis. Nanomaterial-based CDT systems have been shown to achieve a high load of drugs, enhance the enrichment of drugs in tumors, trigger drug release effectively, improve drug efficacy and reduce their systemic toxicity. In the meantime, chemotherapy drugs could also increase H_2_O_2_ concentrations to enhance the effectiveness of CDT.

**Figure 1:**
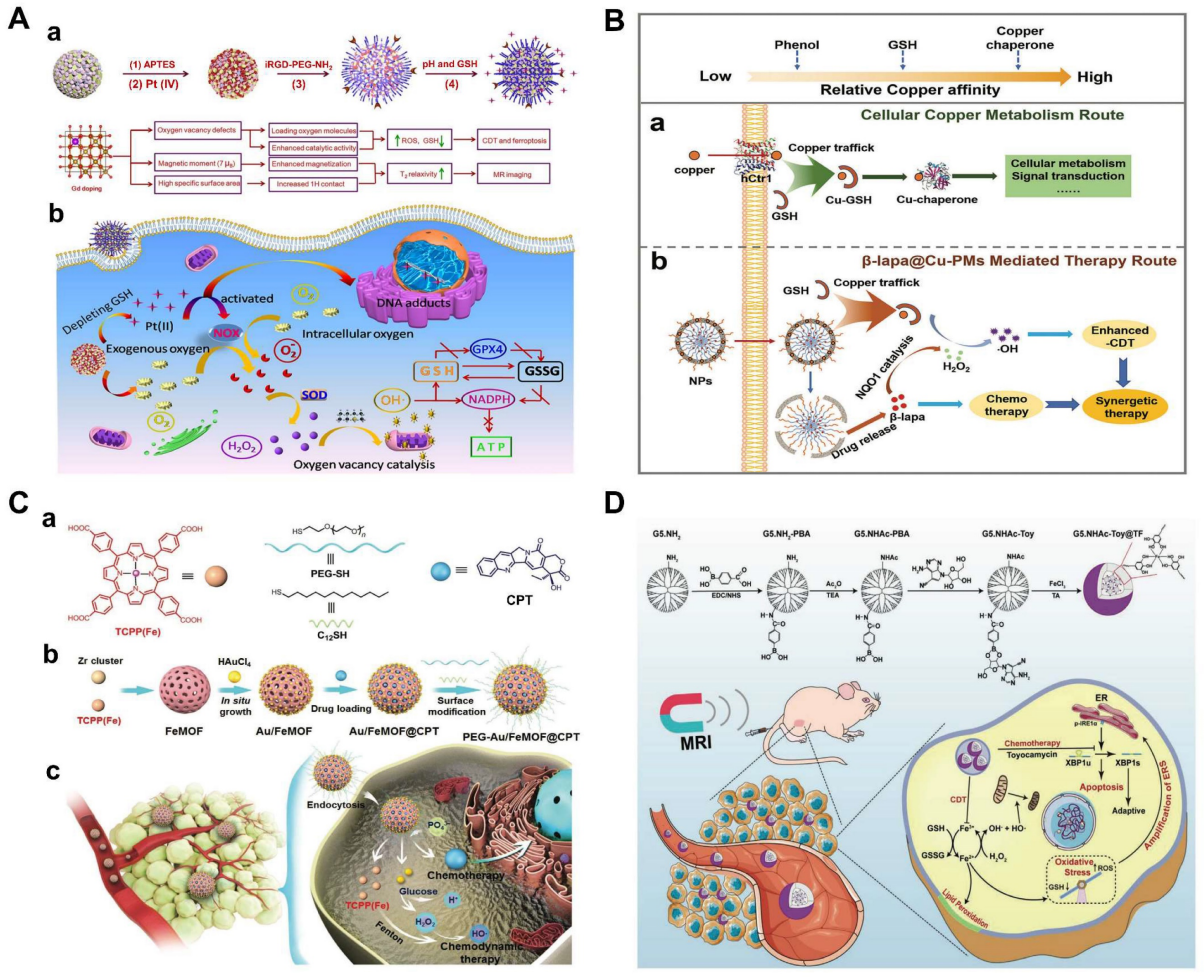
(A) Schematic illustration of the construction of a) iHMNCPt-O_2_ nanoplatform and b) the pathways on which it acts to synergistically accelerate cancer death by combining CDT with chemotherapy. Reproduced with permission 56. Copyright 2020, American Chemical Society. (B) Schematic illustration of a) the intracellular copper metabolism route mediated by GSH and b) the biomimic mechanism of the *β*-lapa@CuPMs therapy route in tumor cells. Reproduced with permission 57. Copyright 2021, American Chemical Society. (C) a) The cartoon illustration and chemical structures of the building blocks (TCPP(Fe), PEG-SH, C_12_SH, and CPT). b) Preparation of the hybrid nanomedicine PEG-Au/FeMOF@CPT. c) High tumor accumulation of PEG-Au/FeMOF@CPT NPs via passive targeting and subsequently cancer cell uptake. Triggered by the intracellular phosphate, the chemotherapeutic drug CPT is released and the cascade catalytic reactions are activated. H_2_O_2_ generated through the oxidation of glucose by Au NPs acts as chemical fuel for Fenton reaction to produce highly toxic ROS to realize chemo/chemodynamic therapy. Reproduced under the terms of the http://creativecommons.org/licenses/by/4.0/ License 59, published by WILEY-VCH Verlag GmbH & Co. KGaA, Weinheim. (D) Schematic illustration of the preparation of G5.NHAc-Toy@TF nanocomplexes for MR imaging and chemotherapy/CDT of tumors in vivo through ERS amplification. Reproduced with permission 61. Copyright 2021, Wiley-VCH.

Doxorubicin (Dox) is commonly used in the treatment of solid tumors and is widely accepted clinically. However, systemic toxicity and multidrug resistance have hampered its clinical application. In addition, Dox may damage normal tissue and cause a range of side effects such as nephrotoxicity, cardiotoxicity, and liver toxicity 44. Developing an effective way to improve the therapeutic effect of Dox in cancer treatment is highly urgent. Metal-organic framework (MOF) is widely used in the medical field and is a promising material for biological application 45,46. MIL-100, a member of the MOFs family, often serves as a carrier of Dox to achieve synergistic CDT/chemotherapy due to its high drug-loading capacity 47-49. Considering the fact that the reaction rate of Fe^2+^ (k = 63-76 M^-1^s^-1^) and the conversion rate of Fe^3+^ to Fe^2+^ (k = 0.001-0.01 M^-1^s^-1^)are slow, Cu ion-based Fenton reagents were developed for Dox carriers to increase hydroxyl radical generation by increasing catalytic efficiency, a novel core-shell metal-organic framework Cu-MOF@SMON/Dox-HA with high drug load, pH/GSH dual responsiveness and good biocompatibility was used for CDT combination chemotherapy 51. In another system, intelligent Dox@Cu_2_O-PEG nanoclusters (NCs) was prepared to enhance CDT/chemotherapy by Cu^+^-mediated Fenton reaction. To increase the stability and compatibility of the NCs, Cu_2_O was externally modified with hydrophilic Polyethylene glycol (PEG) to form Cu_2_O-PEG NCs, which was followed by loading with Dox. The porosity of Cu_2_O-PEG NCs showed the admirable capacity of Dox loading, and the high-density PEG shell-mediated passive action guaranteed efficient accumulation of Dox in the tumor area. What's more, the rapid release of Dox by pH-induced decomposition of Cu_2_O-PEG NCs not only exerts chemotherapeutic effects but also activates nicotinamide adenine dinucleotide phosphate oxidases (NOx) by converting O_2_ into •O_2_^-^ simultaneously, which is further catalyzed by superoxide dismutase to form endogenous H_2_O_2_ to enhance CDT via Cu^+^-mediated Fenton reaction 52. The high expression of GSH and limited H_2_O_2_ content in tumor cells seriously affect the efficiency of tumor treatment. Fortunately, platinum (Pt), β-lapachone (β-lapa), camptothecin (CPT) and bortezomib can activate the NOx in tumor cells and trigger a cascade of reactions that ultimately produce H_2_O_2_ 53-55. In Zhang's study, Fe_3_O_4_ nanoclusters were first rearranged by doping Gd and then self-assembled into hollow magnetic nanoclusters (HMNC), which presented excellent weak acid and GSH responsiveness. The hollow structure could effectively load with platinum (IV) precursor drugs. And a large number of oxygen vacancies could capture oxygen molecules, which enhanced the catalytic activity of HMNC and promotes the production of intracellular ROS. The target agent iRGD-PEG-NH_2_ was then conjugated to the surface of the HMNC-O_2_ to develop iHMNCPt-O_2_ with tumor targeting 56. Platinum (IV) in iHMNCPt-O_2_ would consume GSH in tumor sites and formed platinum (II), which was capable of causing DNA damage and inducing apoptosis. Additionally, iHMNCPt-O_2_ could release oxygen molecules for relieving tumor hypoxia. Subsequently, exogenous and endogenous oxygen molecules were efficiently converted into •O_2_^-^ catalyzed by NO_X_, the increased •O_2_^-^ concentration promoted H_2_O_2_ production to further improve the efficiency of CDT. Besides, the deprivation of GSH and the production of oxygen significantly improved the effectiveness of CDT and chemotherapy. The cascade reactions provide a new method for accelerating the Fenton-like reaction and enhancing the effect of combination therapy (**Figure 1A**).

In another recent study, a GSH-responsive biocompatible nanodrug, called β-lapa@Cu-PMs, was designed 57. The nanodrug combined chemotherapy with chemo-enhanced CDT by coordinating with copper-polydopamine (Cu-PDA) micelles (polystyrene-β-poly(acrylic), PS-b-PAA) micelles, and encapsulated the hydrophobic chemotherapy drug β-lapa. β-lapa@Cu-PMs could achieve copper transfer through GSH response, where copper ions were exchanged from the bicoordination center of PDA phenol (low affinity for Cu(I)) to the thiol site of GSH (high affinity for Cu). After the copper ions escaped, the shell was broken down to release the anticancer drug β-lapa, while β-lapa amplified intracellular H_2_O_2_ levels, further reacted with Cu(I) to generate highly toxic •OH and caused oxidative damage to cells (Figure 1B).

In addition, glucose oxidase (GOD/GOx) has the ability to catalyze the production of H_2_O_2_ by glucose, meeting the requirements for in situ generation of H_2_O_2_. Multifunctional nanomaterials loaded with chemotherapy drugs and GOD are feasible measures to improve synergistic therapy 58. However, uncontrollable reactions between GOx and glucose during delivery and the relatively low operational stability of GOx hinder the practical *in vivo* application of this strategy. Thus, the hybrid nanomaterial Au/Fe MOF NPs with GOx-mimic catalytic activity for CDT/chemotherapy were synthesized by growing Au NPs on the surface of Fe MOF, followed by loading with CPT (**Figure 1C**). The hybridization of Au-NPs greatly improved the stability of nanomedicines in the physiological environment. Attributed to the complex structure, the blood circulation time and tumor accumulation of Au/FeMOF@CPT NPs significantly increased. When Au/FeMOF@CPT NPs were internalized, high concentrations of phosphate within cancer cells triggered their collapse, resulting in complete drug release and activation of cascade catalytic reactions. The Au NPs oxidized glucose to produce H_2_O_2_, which can further work as a chemical fuel for enhanced CDT to achieve the synergistic anticancer effect 59. More importantly, the catalytic activity of Au-NPs could be reasonably controlled by their surface hydrophobicity and subsequent interaction with water soluble glucose so as to specifically activate their enzyme-mimicking catalytic ability in cancer cells, thereby solving the thorny problem of natural GOx for cancer treatment.

Recently, chemotherapy drugs acting on endoplasmic reticulum stress (ERS) may be ideal for combination with CDT to achieve enhanced combined antitumor therapy 55. Toyocamycin (Toy), as a promising anti-cancer drug, can prevent IRE1α splicing X-box binding protein 1 (XBP1u) to effectively inhibit the survival of cancer cells 60. In the latest study, a dendritic polymer-drug conjugate using tannic acid (TA) chelated Fe^3+^ was used for MR imaging-guided tumor CDT/chemotherapy by ERS amplification 61. First, G5 poly(amidoamine) (PAMAM) dendritic macromolecules with amine terminations (G5.NH_2_) were linked to PBA, acetylated to neutralize the remaining terminal amines (G5.NHAc-PBA), and covalently coupled with Toy by borate ester bonds to obtain the G5.NHAc-Toy@TF complex. The G5. NHAc-Toy@TF nanomedicines had good stability, responsive drug release behavior, and could generate cytotoxic hydroxyl radicals through the Fenton reaction, thereby amplifying ERS for improved CDT/chemotherapy of cancer cells *in vitro* and xenograft breast tumor models *in vivo* (**Figure 1D**).

Many other CDT-like agents and chemotherapeutic drugs have also been explored for synergistic treatment. Artesunate (ASA) is a semi-synthetic derivative of artemisinin and is commonly used as an antimalarial agent 62. In Hou's work, Methotrexate (MTX), gadolinium (Gd) and ASA have been incorporated into NPs, which could be activated by tumor-specific endogenous Fe^II^ for ROS amplification and enhancing CDT/chemotherapy. ASA-MTX-Gd^III^ NPs were specifically endocytosed by tumor cells. The released ASA could be catalyzed by tumor-specific overexpressed endogenous Fe^II^ ions and produce enough ROS to enhance the CDT, which enabled a synergistic effect with the adjuvant chemotherapy of MTX. It is worth mentioning that the highly toxic ROS induced by tumor-specific endogenous stimulation could avoid the use of exogenous conditions and the limitations of severe Fenton conditions 63. Furthermore, vorinostat has also been used to synergize with the CDT by being encapsulated into CuNPs through strong coordination with copper ions. Vorinostat can inhibit the activity of histone deacetylases (HDACs) overexpressed in tumor cells to achieve the purpose of synergistic cancer treatment 64. Apart from the above substances, parthenolide, tirapazamine, and other chemotherapeutic drugs have been explored to cooperate with Fenton reaction-mediated CDT to enhance the therapeutic effect 65, 66.

In short, the combination of CDT and chemotherapy has a significant synergistic effect, which not only enhances the efficacy of CDT by increasing the concentration of H_2_O_2_ by chemotherapy drugs, but also reduces the side effects of drugs. It enables drugs to effectively spread within tumors, improves drug bioavailability and circulating half-life *in vivo*. However, the relatively low drug loading and slow release of Fenton reagent may weaken its anti-tumor effects, and systemic side effects caused by chemotherapy drugs are difficult to be completely suppressed, and newly introduced nanocarriers might further increase side effects. The development of carrier-free nanomedicines, or “non-toxic to toxic” transformation caused by microenvironmental stimulation, or nanomedicines constructed from organic materials with high biocompatibility, good biodegradability, and high drug load, may be the way forward. In addition, the elevated H_2_O_2_ concentration caused by chemotherapy drugs is limited both the electron donor and the oxygen content of the tumor. In hypoxic regions, it isn't easy to produce sufficiently high concentrations of H_2_O_2_.

### CDT in Combination with PDT

PDT has been explored as a minimally invasive treatment modality for oncological and non-oncological diseases, which relies on specific wavelength of light irradiation to activate photosensitizers to produce biotoxic singlet oxygen 67. PDT is one of the typical ROS-involved antitumor therapies, which mainly relies on photosensitizers such as porphyrins, chlorins derivatives, or photoactive nanoparticls (NPs) to generate ROS by electron transfer or energy transfer under external light to induce apoptosis and necrosis. PDT has many advantages, including non-invasive, low side effects, and low systemic toxicity 68, 69. However, several drawbacks, such as oxygen dependence and limited light penetration depth, still limit ROS production and compromise the therapeutic results for conventional PDT.

**Figure 2:**
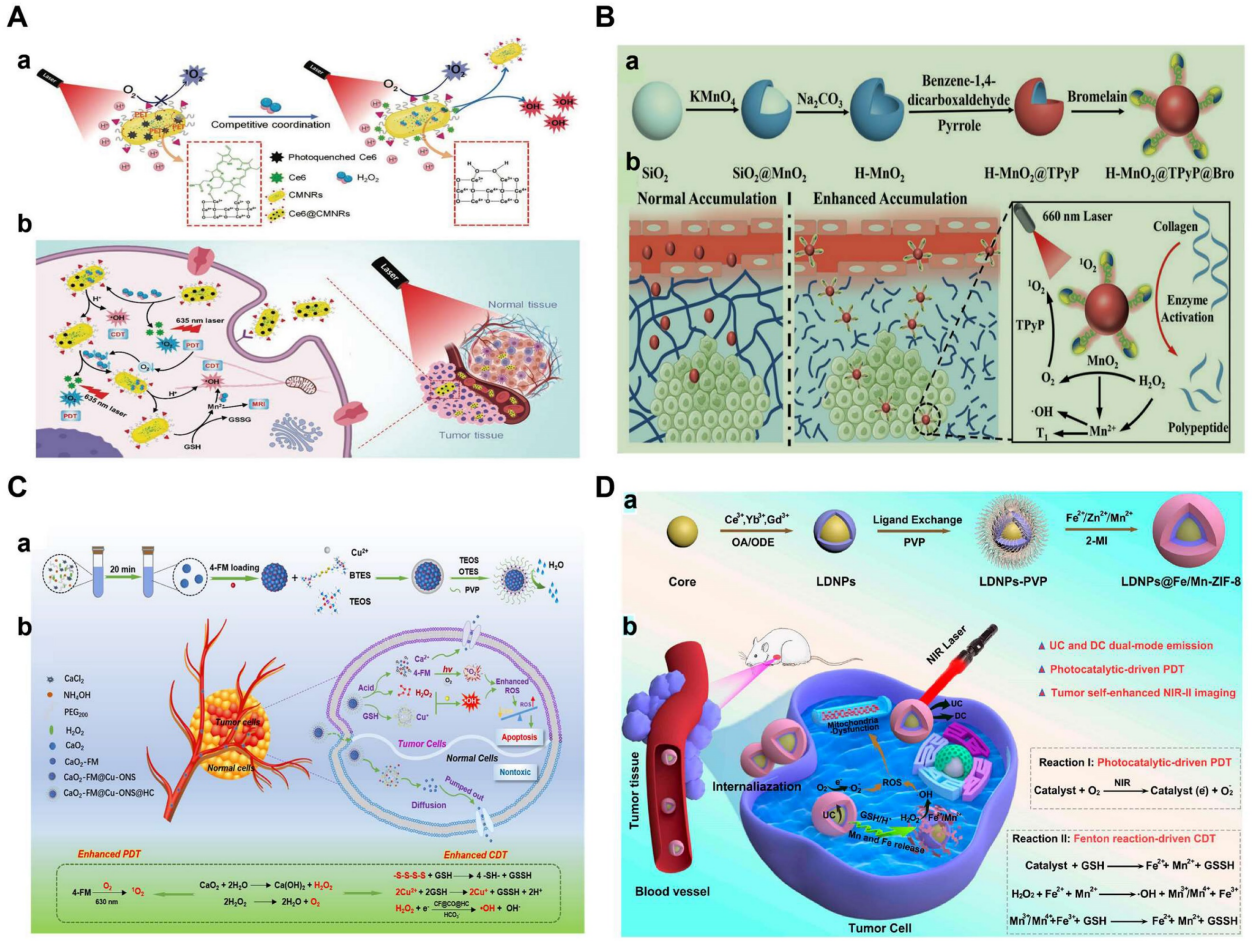
(A) a) Schematic mechanism of the competitive coordination process for the CeO_2_-MnO_2_ nanozyme. b) Schematic illustration of Ce6@CMNRs for tumor specific synchronously activated combination therapy under the guidance of MRI. Reproduced with permission 69. Copyright 2022, Wiley-VCH. (B) a) Schematic illustration for the synthesis of H-MnO_2_@TPyP@Bro. b) Enzyme activation of H-MnO_2_@TPyP@Bro toward collagen digestion for enhanced accumulation of nanoparticles in the tumor 77. Copyright 2022, Wiley-VCH. (C) Schematic of a) preparation process and b) therapeutic mechanism of CF@CO@HC for PDT/CDT synergistic therapy. Reproduced with permission 78. Copyright 2022, American Chemical Society. (D) Schematic diagram for the preparation of LDNPs@Fe/Mn-ZIF-8 a) and the mechanism of catalytic therapy and NIR-II imaging of LDNPs@Fe/Mn-ZIF-8 in the TME under NIR laser irradiation (b). Reproduced with permission 84. Copyright 2022, American Chemical Society.

The combined therapy of PDT and CDT is therefore important and imperative to overcome the limitations of single treatment. Chlorine e6 (Ce6), as a representative photosensitizer, is a suitable chlorine derivative for the PDT 70, 71. However, the poor water solubility prevents the application of Ce6. Aimed to this, a MnO_2_-doped CeO_2_ nanozyme-based nanomedicine (Ce6@CMNRs) was reported to load with Ce6 for tumor-specific synchronous activation of CDT/PDT 69. Tumor overexpressed H_2_O_2_ replaced Ce6 on the Ce6@CMNRs surface by competing for coordination and decomposed into •OH under acidic conditions to achieve CDT. At the same time, the substituted Ce6 triggered the PDT under laser irradiation, which was inhibited before the replacement occurred. In addition, activated PDT-induced oxygen deficiency further induced the production of H_2_O_2_ to constantly replace the residual Ce6 of surface coordination, resulting in the complete activation of PDT and CDT (**Figure 2A**). More importantly, due to the property of CeO nanorods, various molecules could coordinate to their surface and be replaced by H_2_O_2_ through competitive coordination. Therefore, other kinds of photosensitizers or chemotherapeutic drugs can also be selected to coordinate on the surface of CeO_2_-MnO_2_ nanorods to achieve different types of combined treatment and imaging modes. In addition, other Fenton reagent-based materials were also used as carriers for Ce6 to enable enhanced synergistic therapy. For examples, hollow structured manganese carbonate (MnCO_3_) nanocubes or MIL-100 NPs could ensure efficient Ce6 loading for CDT/PDT 72,73. Additionally, the assembly of Ce6-modified carbon dots and Cu^2+^ could restore PDT under the stimulation of TME, which avoided the phototoxicity of photosensitizers in normal tissues 74.

Notoriously, the hypoxic state and high expression of GSH seriously affect the effect of PDT or CDT. Based on this, various reagents for O_2_ release and GSH consumption have been developed 75, 76. In order to improve the enrichment of photosensitizers in the deep tumor and ameliorate the hypoxia of the TME, a multifunctional hollow PDT nanoplatform (H-MnO2@TPyP@Bro) composed of MnO_2_, porphyrin (TPyP) and bromelain (Bro) was successfully designed 77. In this system, Bro digested collagen, thereby enhancing H-MnO2@TPyP@Bro enrichment in deeper areas of the tumor. MnO_2_ not only reacted with GSH, released Mn^2+^ ions and subsequently catalyzed the conversion of H_2_O_2_ to •OH to achieve CDT, but also catalyzed H_2_O_2_ to produce O_2_ in situ, reducing tumor hypoxia and thus improving PDT. It is worth noting that the hollow nanostructures of MnO_2_@TPyP@Bro could improve the utilization rate of light through multiple reflections and maximize the effect of PDT. In addition, the released Mn^2+^ could activate MRI to supervise the treatment process (**Figure 2B**). H-MnO_2_@TPyP@Bro in combination with CDT and PDT has great potential in the treatment of hypoxic tumors.

In another recent study, an O_2_/H_2_O_2_ self-sufficient nanodrug CaO_2_-FM@Cu-ONS@HC (CF@CO@HC) was designed for enhanced CDT/PDT combination therapy. CaO_2_ NPs were first prepared, then loaded with a high-efficiency photosensitizer 4-DCF-MPYM (4-FM), followed by CaO_2_ FM cores encapsulated with Cu^2+^ and tetrasulfide bond-doped smart silicone shells (Cu-ONS) (**Figure 2C**). After endocytosis into 4T1 tumor cells, Cu^2+^ and tetrasulfide bonds could be reduced by high concentrations of GSH. The exposed CaO_2_ was then biodegraded to produce H_2_O_2_ and O_2_ and release photosensitizers at the same time. Taken together, CF@CO@CH overcame the barriers of tumor hypoxia in PDT and H_2_O_2_ deficiency in CDT. Besides, GSH depletion capacity further improved the therapeutic effect of ROS-mediated CDT/PDT synergistic therapy 78.

Apart from the above photosensitizers, FeTCPP/Fe_2_O_3_ NPs (organic framework nanoparticles based on tetra (4-carboxyphenyl) porphyrin) with peroxidase activity that catalyze H_2_O_2_ to form^ 1^O_2_ 79, ultra-thin two-dimensional metal-organic frameworks Cu-TCPP composed of Cu^2+^ and TCPP ligands that selectively produce ^1^O_2_ in the TME 80, and 1.2-[1-hexyloxyethyl]-2-divinyl pyrophosphate-a (HPPH), a second-generation photosensitizer with significantly reduced phototoxicity 81 have also been used in the combined therapy of CDT and PDT.

However, conventional organic photosensitizers suffer from poor stability, low solubility, low loading capacity, and early release from the carrier, which seriously prevent further clinical applications of PDT. Recently, semiconductors, photocatalysts and metal-organic framework (MOF)-based nanocomposites (ZIF-8) have been considered as potential photosensitizers due to their high photostability, good biocompatibility, and wide photo-response ranging from UV to visible light and used to achieve CDT by reacting with the overexpression of H_2_O_2_ and GSH 82, 83. ZIF-8 has broad application prospects in cancer diagnosis and treatment because of its rich physicochemical properties. However, due to the wide band gap and specific response to ultraviolet light, the photocatalytic anti-cancer application of ZIF-8 is still limited. In view of this, Prof. Yang et al. developed lanthanide-doped nanoparticles (LDNPs) doped with Fe/Mn bimetals and coated with ZIF-8 (LDNPs@Fe /Mn-ZIF-8) for NIR-II imaging-guided CDT/PDT 84. LDNPs@Fe /Mn-ZIF-8 can achieve dual-mode red upconversion (UC) and NIR-II downconversion (DC) emission under NIR laser irradiation. At the optimal doping concentration (concentration of Ce^3+^ was 20%), the emission intensity of UC and DC NIR-II of LDNPs was 30.2 and 13.2 times higher than that of pure nuclear nanoparticles, respectively, which also made LDNPs@Fe/Mn-ZIF-8 with excellent UC-mediated PDT and NIR-II optical imaging capabilities. In addition, the double doping of Fe^2+^/Mn^2+^ significantly reduced the band gap (5.1 eV to 1.7 eV) of the ZIF-8 photosensitizer and expanded its excitation threshold to the visible region (650 nm). Therefore, Fe/Mn-ZIF-8 was effectively excited by UC photons to achieve photocatalytically-driven PDT. Furthermore, Fe/MnZIF-8 could also be degraded in the TME to release Fe^2+^/Mn^2+^ ions, which in turn produced •OH through Fenton-like reactions to implement CDT. At the same time, the degradation of Fe/Mn-ZIF-8 also equipped nanosystem with self-enhanced tumor NIR-II imaging capabilities, which provided precise guidance for CDT/PDT (**Figure 2D**).

Besides, in order to overcome the undesired damage to normal tissue caused by the non-specific uptake of photosensitizers, the receptor-mediated pathway was applied to precisely deliver photosensitizers to the cancer site. In one nanosystem, HA-Ru nanoaggregates (NAs) were hybridized from Cys-HA and Ru NPs, which rationally integrated receptor-mediated targeting (RMT) and tumor-microenvironment responsiveness for enhanced cancer phototherapy. HA components endowed HA-Ru NAs with RMT properties to selectively identify CD44-overexpressed cancer cells, which would effectively improve the specificity and efficacy of phototherapy. And Ru-NPs were considered to be “smart” Ru-based nanozymes that achieved H_2_O_2_-responsive therapy for the first time. Ru NPs not only exhibited NIR-mediated photothermal and photodynamic functions, but also possessed catalytic enzymatic and peroxidase-like activities that could catalyze the H_2_O_2_ to O_2_ for tumor hypoxia relief and toxic •OH for CDT. Meanwhile, Ru-based nanomaterials with PAI and CT capabilities were also used for tumor diagnosis, enabling the integration of tumor diagnosis and treatment 85. Besides, phthalocyanine photosensitizers, such as ZnPc, have also been explored for CDT/PDT antitumor therapy 86.

In conclusion, the combination of CDT/PDT is mainly achieved by loading the photosensitizer on the Fenton reagent or coordinating the self-assembly of transition metal ions with the photosensitizer, which can increase the concentration of ROS in TME under photoexcitation. The efficacy of PDT depends on O_2_ and laser, however, the hypoxic environment and limited depth of light penetration limit the efficiency of ROS generation during PDT. A combination of PDT and CDT is an effective strategy to increase ROS content and activity. However, High levels of GSH, the hypoxic environment and low concentrations of H_2_O_2_ in cancer cells are the main barriers to PDT and CDT. In order to maximize the therapeutic effect of PDT and CDT, Photosensitizers with the Fenton effect that can reduce intracellular GSH levels, ameliorate hypoxia, or increase intracellular H_2_O_2_ levels need to be further developed. At the same time, the phototoxicity of photosensitizers, the poor tumor enrichment effect of small molecule photosensitizers, the lower quantum yield of inorganic photosensitizers, and the limited maximum penetration depth of light are all fatal shortcomings affecting the efficiency of this combination therapy.

### CDT in Combination with PTT

PTT is a minimally invasive tumor treatment technology, which uses photothermal conversion agents (PTAs) to absorb near-infrared light and converts the absorbed light energy into heat so that the temperature of the tumor site rises, thereby inducing apoptosis of tumor cells 87. However, PTT alone is less effective in deep tumors due to the limited tissue penetration of light. A combination of CDT and PTT is a viable way to improve treatment outcomes. Some PTAs with inherent peroxidase could be designed to enhance the CDT effect by encapsulating Fenton reagents. Additionally, PTAs with the Fenton effect or Fenton-like effect can solve the contradiction between photothermal conversion efficiency and biodegradability because PTAs with catalytic ability are usually able to respond to TME, which makes them with slow degradation and little loss of photothermal conversion ability 88. What's more, the increased temperature will accelerate the chemical reaction kinetics, resulting in a higher reaction rate and product yield per unit of time. Studies have shown that the local temperature rise induced by near-infrared light significantly accelerated the Fenton reaction in the tumor, which could improve the therapeutic effect of CDT 34.

**Figure 3:**
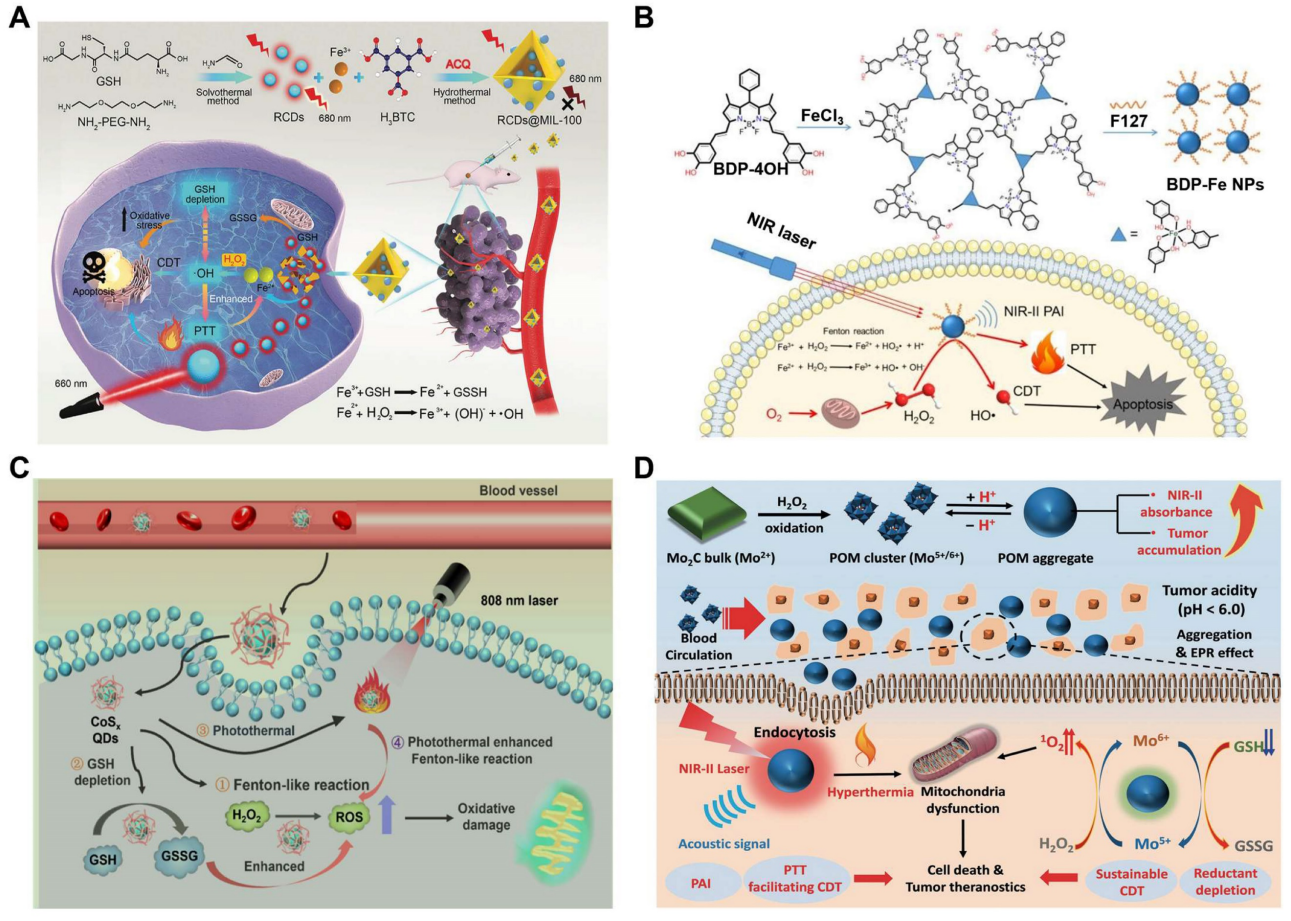
(A) Schematic representation of the principle of the RCDs@MIL-100 nanoplatform for TME-activated cancer imaging and chemodynamic photothermal combined therapy. The ACQ is abbreviation for aggregation-caused quenching. Reproduced with permission 89. Copyright 2022, Wiley-VCH. (B) Synthetic route and schematic diagram of BDP-Fe NPs for the synergistic PTT and CDT. Reproduced with permission 94. Copyright 2020, The Royal Society of Chemistry. (C) Schematic illustration of biodegradable CoSx QDs for PTT and hyperthermal-enhanced CDT of tumors. Reproduced with permission 100. Copyright 2022, American Chemical Society. (D) Illustration of synthesis and working mechanisms of the POM. Reproduced with permission 101. Copyright 2019, Wiley-VCH.

Large numbers of nanotherapeutics such as small organic molecules (e.g. porphyrin, boron dipyrrole (BODIPY), phthalocyanine and croconaine), transition metal-based materials, carbon materials, noble-metal nanostructures, and semiconductor polymers have been designed for synergistic PTT and CDT in cancer treatment. MOF materials have attracted widespread attention in CDT/PTT combination therapy due to their abundant active sites and relatively large surface area. One recent study reported that MIL-100 MOF as a photothermal material carrier and TME stimuli-responsive nanomedicines for CDT/PTT has been reported. In this study, near-infrared emitting carbon dots (RCDs) were initially prepared using GSH as the precursor. They had strong photothermal conversion ability under 660nm laser irradiation, which could be used for efficient PTT (**Figure 3A**). The RCDs@MIL-100 was then self-assembled by hydrothermal method using RCD, FeCl_3_, and trimellitic acid solutions 89. In TME, RCDs@MIL-100 consumed the amount of GSH and increased the concentration of Fe^2+^, then the released Fe^2+^ reacted with H_2_O_2_ to produce •OH for CDT. Moreover, increased temperature at the tumor site promoted RCDs@MIL-100 and GSH redox reactions, accelerated the Fenton reaction between Fe^2+^ and H_2_O_2_, induced oxidative stress amplification, and improved the thermal sensitivity of cancer cells. On the other hand, the released small-sized RCD efficiently entered the deep tissues of the tumor to improve the efficiency of the PTT and switched from the “off” state of quenching caused by aggregation to the “on” state of fluorescence recovery, thus enabling fluorescence imaging of tumor tissue. Therefore, GSH depletion and high temperature induced by photothermal heating intensified the tumor-specific oxidative stress amplification, and the effective synergy of CDT and PTT was practical under the guidance of near-infrared fluorescence imaging. In another study, Dai's group used polyethyleneimine (PEI)-coated Fe_3_O_4_ to react with PEGylated multiwalled carbon nanotubes (MWNTs), and then combined with GO_X_. The as-obtained therapeutic agent could convert glucose into H_2_O_2_ and gluconic acid, which would enhance the Fenton reaction catalyzed by Fe_3_O_4_ nanoparticles. Besides, the mild heat accelerated the chemical reaction kinetics, which produced more •OH per unit time and enhanced the efficacy of CDT/PTT 90.

In recent years, significant progress has been made in the optimal design of small organic molecule photothermal agents to improve their photophysical and photochemical properties. Nanoscale coordination polymers (NCPs) formed by metal and organic ligands have attracted significant attention in the Fenton reaction. The separated metal centers could endow NCP with rich catalytic sites and excellent TME response properties. Meanwhile, it also served as an electron transfer medium to tune the type of ROS. Furthermore, the 3D structure of NCPs linked by rigid ligands formed a large number of mesopores, which would increase the probability of contact between the active substrate (e.g., H_2_O_2_, O_2_, H_2_O) and the metal or ligand, and accelerate the generation of ROS 91. And the introduction of photoactive ligands greatly contributed to NCP phototherapy. The photoactive ligands of NCP are mainly concentrated on porphyrin derivatives, such as indocyanine green and IR825. However, NIR-I (usually 808nm) absorption of cyanine dyes limited their application 92. Compared with the commonly used NIR-I light source, NIR-II (1000nm-1700nm) provided a better tissue penetration for deep tumor treatment 93. Thus, three-dimensional boron dipyrrole ethylene (BODIPY)-Fe^3+^ coordination polymer nanoparticles (BDP-NPs), which could passively target tumor sites through the EPR effect, have been developed 94. Benefiting from the 3D structure of BDP-Fe NPs, H_2_O_2_ could more easily enter the NPs, interact with Fe^3+^, and remain stable in TME, which was further catalyzed to produce •OH. More importantly, BDP-NPs can extend the light absorption to 1300 nm with higher photothermal conversion, exhibiting synergistic chemodynamic/photothermal therapy (**Figure 3B**).

In addition to carbon-based materials and small molecules, transition metal-based materials with specific structures have shown great potential in PTT due to their strong NIR absorption, excellent photothermal conversion efficiency, and magnetism 95, 96. Chen et al. first synthesized a novel subminiature bovine serum albumin (BSA)-CuFeS_2_ NPs using biomineralization strategy. They used BSA as a template and bonded copper and iron ions through the excellent affinity of carboxyl and surfactant to enhance its biocompatibility 97. The BSA-CuFeS_2_ NPs exhibited unique pH-independent Fenton-like reaction characteristics that could efficiently generate •OH in a slightly acidic tumor environment. Additionally, BSA-CuFeS_2_ NPs had good photothermal conversion efficiency for PTT and for enhancing CDT. In addition, the ultra-small size gave BSA-CuFeS_2_ NPs the ability to quickly excrete from the body through the kidney and liver to avoid long-term and systematic toxicity effectively. *in vivo* and* in vitro* experiments showed that BSA-CuFeS_2_ NPs had no apparent toxicity and could significantly enhance the therapeutic outcome of CDT/PTT. Another study synthesized the photothermal Fenton nanocatalyst (PFN) by incorporating MnO_2_, CuS, and HSA in the acidic TME for NIR-II guided PTT and CDT 98. Furthermore, a new two-dimensional (2D) FPS-PVP platform with high NIR-II photothermal conversion efficiency (up to 43.3%) and superior Fenton catalytic activity based on biocompatible FePS_3_ (called FPS) nanosheets have also been developed for CDT/PTT as well 99.

Transition metal dichalcogenide (TMD) quantum dots (QDs), such as cobalt sulfide (CoSx), have attracted some attention due to their advantages such as atomic-scale thickness, direct band gap, and good electronic properties. The inherent polyvalence in Co, combined with the wide absorption range in the NIR window, makes CoSx a potential candidate for PTT/CDT therapeutics. In order to precisely control the photothermal conversion efficiency (PCE) and Fenton-like activity of CoSx ODs, Professor Zhu's group has devised a simple and gentle strategy to synthesize a series of biocompatible CoSx ODs with varying degrees of defects for the first time to optimize CDT and PTT 100. To study defect engineering, CoSx QDs with different sulfur defects were prepared by varying the initial feed molar ratios of Co^2+^ and S^2-^ (1:0.5, 1:1, 1:2, 1:4, 1:6, and 1:8 and named them CoSx ODs 1:0.5, CoSx ODs 1:1, CoSx QDs 1:2, CoSx-QDs 1:4, CoSx- ODs 1:6, and CoSx ODs 1:8). After a series of experiments, it was found that the CoSx QDs 1:2 group showed relatively high PCE and methylene blue degradation rates, so the synergistic therapeutic effect of CoSx QDs 1:2 may be higher than that of other sulfur level groups. When CoSx QDs were endocytosed by cells, they could induce cancer cell death in four processes simultaneously: (1) a Fenton-like reaction, CoSx QDs catalyzed endogenous H_2_O_2_ to produce ROS; (2) converting GSH to GSSG through redox reactions, disrupting the cellular antioxidant defense system; (3) ablation of cancer cells by PTT of CoSx QDs; and (4) heat generation increased the Fenton reaction rate between CoSx QDs and H_2_O_2_. In addition, the degradability and low toxicity of CoSx QDs may lead to efficient metabolism and clearance with good biocompatibility and biosafety (**Figure 3C**). Therefore, defect-driven CoSx QDs could be used as a tumor-specific multifunctional drug with flexible and controllable efficacy in future cancer treatment.

Many other transition metal materials, such as molybdenum and tungsten, have also been developed as combined therapeutic agents for photothermal and chemodynamic therapy. Molybdenum-based (Mo^5+^/^6+^) nanotherapeutics are of great interest since the reduced Mo^5+^ ions make PTT and PAI possible via internal photo-induced charge transfer. A typical example here was Mo_2_C-derived polyoxometalate (POM) clusters prepared by self-assembling in mild acidity, which enhanced NIR-II absorption and provided better tissue permeability for deep tumor treatment 101. The POM would accumulate in tumors due to the enhanced permeability and retention (EPR) effect, then MO^5+^ was oxidized to Mo^6+^, which could produce a large amount of toxic ^1^O_2_. Enhanced CDT efficiency was also attributed to the POM's photothermal properties and GSH depletion ability. Under the *in vivo* guidance of PAI, CDT/PTT combination therapy showed excellent effects in tumor ablation and prevention of tumor recurrence (**Figure 3D**).

Similarity, tungsten also has been studied for its biological applications 102. Tungsten oxide is a common photocatalyst for the degradation of organic dyes. Considering the presence of W^5+^ and W^6+^ valence states in WO_3_, it also shows excellent potential as the Fenton-like reagent. Despite this, limited studies have investigated the potential CDT application of WO_3_, and the photothermal enhancement of CDT even gets less attention. A previous study showed that ultrasmall WO_3_-x@γ-PGA NPs were prepared for PAI-guided synergistic PTT/CDT therapy. The obtained WO_3_-x@γ-PGA NPs exhibited good NIR-II photothermal properties. They utilized mild heat to promote the efficacy of CDT, demonstrating photothermally enhanced CDT effects both *in vitro* and *in vivo* 103. Notably, this study used mild PTT to amplify the Fenton reaction and achieved a high therapeutic effect. Meanwhile, mild PTT can effectively avoid collateral damage to adjacent healthy cells due to heat diffusion.

Therefore, the combination of CDT and PTT can produce a significant synergistic effect. The increased temperature not only accelerates the catalytic efficiency of the Fenton/Fenton-like reaction, but also promote blood circulation and increase oxygen at the tumor site, constructing a microenvironment more suitable for CDT. And it is worth noting that CDT can improve the heat sensitivity of tumor cells through oxidative stress induction. The fatal disadvantage of PTT in the treatment process is the low penetration depth in the body, which significantly limits the application of PTT, and the local hyperthermia of the tumor caused by PTT may damage normal tissue. To overcome the issue of light penetration in the body, the researchers used ultrasound instead of lasers. In addition, the NIR-II window can provide more robust tissue penetration depth. Thus, the combination of NIR-II laser-mediated PTT and CDT could achieve better treatment outcomes. With the further improvement of optical transmission technology, such as built-in optical fibers or wireless photons, this combination will be a promising treatment for removing deeper tumors.

### CDT in Combination with SDT

Ultrasound (US), a non-invasive sound wave with high tissue penetration depth, has a wide range of biomedical applications, i.e., drug release control, tumor eradication, and SDT 104. SDT is a novel treatment modality that makes use of ultrasound stimulation to activate sonosensitizers to produce ROS, cavitation, bubbles, heat, etc. 105, 106. Benefiting from the deep tissue penetration, SDT is more suitable for clinical applications than PDT 107. However, hypoxia and excess GSH hinder the effectiveness of SDT alone 108. CDT/SDT combination therapy could significantly improve the efficiency of tumor treatment by increasing ROS production. In addition, a recent study has found that the introduction of US can enhance the activity of the Fenton reaction and directly improve the efficiency of CDT 109. Due to the great clinical potential of SDT/CDT in treatment of deep tumors, it is considered as the development direction of bimodal therapy.

**Figure 4:**
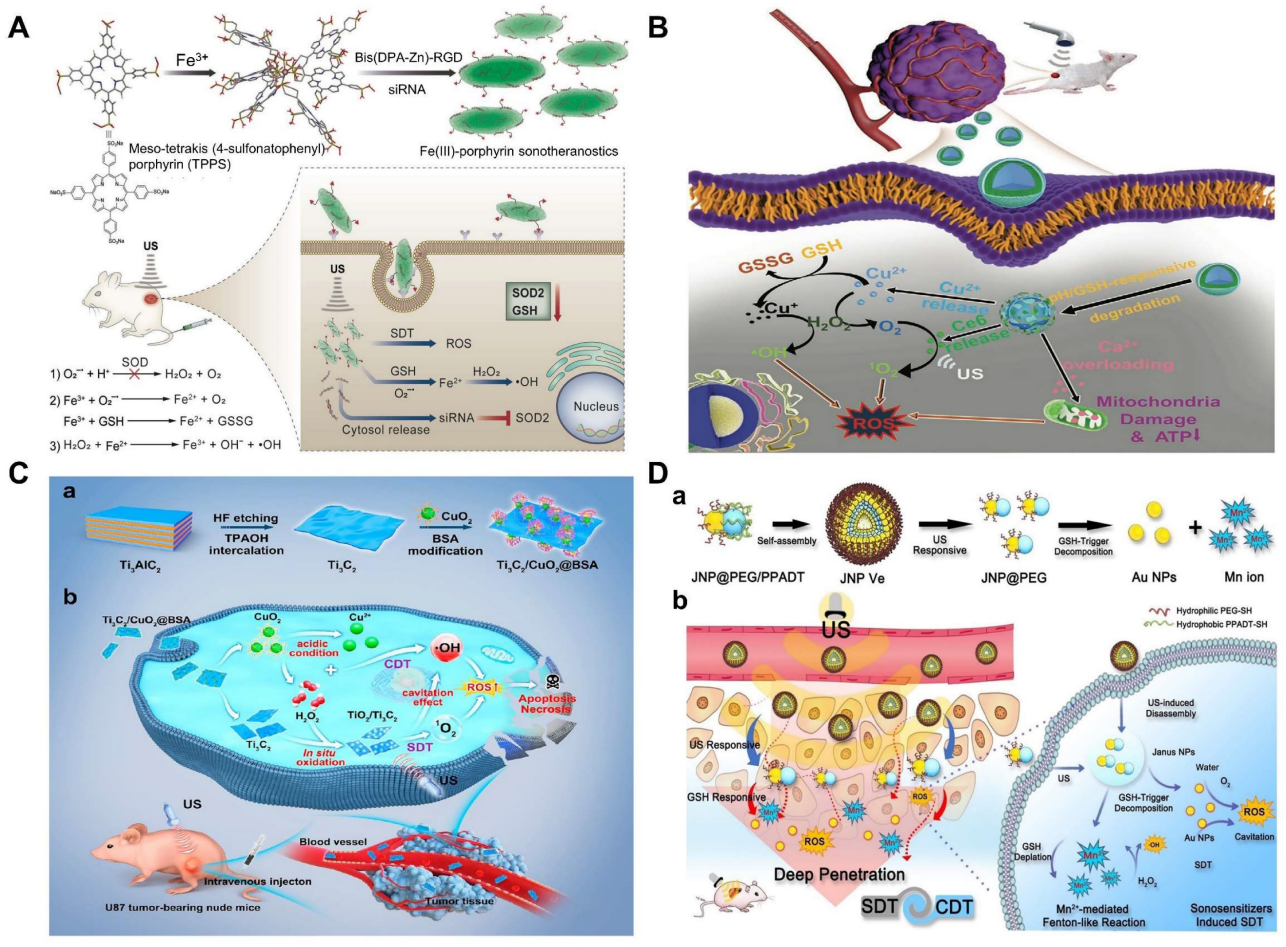
(A) Schematic illustration of the R-S-NTP serving as a multifunctional sonotheranostics. This sonotheranostics was fabricated via coordinating Fe (III) and TPPS, followed by anchoring with Bis (DPA-Zn)-RGD and manganese superoxide dismutase (SOD_2_)-siRNA. The R-S-NTP could substantially accumulate in the tumor via EPR effect and the active tumor-targeting ability of RGD. Upon uptake by cancer cells, the R-S-NTP could specifically down-regulate SOD_2_ expression, trigger GSH depletion and Fenton reaction generation, and thus achieve highly efficient anticancer therapy. Reproduced with permission 113. Copyright 2019, Wiley-VCH. (B) Cu/CaCO_3_@Ce6 nanoparticles (CCC NPs)-dominated advanced cancer therapy via multiple reactive oxidative species (ROS) amplification 114. Copyright 2022, Wiley-VCH. (C) Schematic illustration of 2D MXene-originated in situ nanosonosensitizer generation for augmented and synergistic sonodynamic tumor nanotherapy. a) Schematic illustration of the synthesis of Ti_3_C_2_/CuO_2_@BSA nanosheets. (b) Synergetic chemodynamic and sonodynamic therapeutic process of 2D Ti_3_C_2_/CuO_2_@BSA nanosheets under US irradiation. Reproduced with permission 118. Copyright 2022, American Chemical Society. (D) Illustration of a) self-assembly of amphiphilic Janus Au-MnO NPs into functional vesicles and b) their sequential US and GSH-induced disassembly into small JNPs with deep tumor penetration for synergistic SDT/CDT. Reproduced with permission 121. Copyright 2020, Wiley-VCH.

There is no doubt that ultrasound-activated sensitizers play an important role in SDT. From the early organic molecules to the current inorganic sonosensitizers, the use of sonosensitizers in SDT has been shown to be effective in enhancing therapeutic outcomes. Traditional sonosensitizers are mainly organic molecules, i.e., porphyrins, chlorophylls, curcumin and Ce6 110. These organic molecules could be activated upon ultrasound exposure from the ground to the excited state, leading to ROS production 111. Porphyrin and its derivatives are the most used sonosensitizers in SDT and have been extensively studied. However, these small porphyrins are less chemically and biologically stable and require further optimization. A series of recent studies have found that metalloporphyrin complexes can optimize these properties 112. For instance, Liu's group had designed a multifunctional nanoplatform (named R-S-NTP) based on Fe (III), meso-tetrakis (4-sulfonatophenyl) porphyrin (TPPS), Bis (DPA-Zn)-RGD, and manganese superoxide dismutase (SOD_2_, an important antioxidant enzyme) siRNA for enhancing the production of ROS through targeted delivery and triple regulation methods, including SOD_2_ down-regulation, glutathione depletion and Fenton reaction (**Figure 4A**). In this smart system, sonosensitizer TPPS had high ROS generation and could be assembled with Fe (III) to obtain Fe (III)/TPPS nanostructures. Bis (DPA-Zn)-RGD not only imparted the ability of Fe (III)/TPPS to target active tumor but also acted as a vector for siRNA delivery. After R-S-NTP was internalized by cells, the released Fe^3+^ reacted with GSH, and the subsequent depletion of GSH thus enhanced the Fenton reaction and SDT. SOD_2_ siRNA downregulated SOD_2_ expression, resulting in a significant increase in the production of •O_2_^-^ and Fe^2+^, which provided a steady stream of raw materials for the Fenton reaction and further strengthened the CDT. Furthermore, cells treated with R-S-NTP+US showed higher ROS production compared to the control groups. These results indicated that R-S-NTP exhibited excellent cell uptake, reduced antioxidant capacity, and enhanced CDT and SDT 113. However, nanotherapeutics prepared by chelating organic sonosensitizers with Fenton ions are limited by the low water solubility of chelates and the poor stability during ultrasonic treatment. Therefore, it is necessary to develop stable carriers to load sonosensitizer.

As an O_2_-dependent treatment, SDT will aggravate tumor hypoxia and greatly inhibit its effect. Therefore, O_2_ should also be a breakthrough to improve the efficiency of the synergistic treatment. Calcium (Ca^2+^) overload-mediated mitochondrial membrane potential decreases, causing mitochondrial breathing disorders, which in turn trigger ROS outbreaks. Thus, the fabrication of calcium-based NPs with tumor-responsive Ca^2+^ release causes persistent mitochondrial dysfunction, which could be a new strategy for the treatment of malignant tumors. In Professor Lin's work, a Cu-containing TME response decomposition CaCO_3_ nanoplatform loaded with Ce6 was constructed (Cu/CaCO_3_@Ce6, or CCC NPs) (**Figure 4B**). CCC NPs released Ca^2+^, Cu^2+^ and Ce6 under the action of weak acids and GSH. Cu^+^-mediated Fenton reaction was used for CDT. At the same time, the influx of Ca^2+^ led to mitochondrial dysfunction and intensified oxidative stress. Moreover, Ce6 in tumor cells produced a large amount of ^1^O_2_ under US irradiation for SDT. Subsequently, with the help of H_2_O_2_, continuous Cu^2+^ influx induced the generation of O_2_ to alleviate the hypoxia in solid tumors, which in turn enhanced SDT 114. The platform integrates oxygen self-supply, GSH deprivation, and ROS amplification to achieve the synergistic CDT/SDT treatment effect of 4T1-bearing mice.

In the past five years, the development of inorganic sonosensitizers for CDT/SDT has rapidly increased, such as titanium dioxide NPs, gold NPs, graphene, and mesoporous silica. Compared with organic sonosensitizers, they have superior physical and chemical properties and stability 115, 116. Pure TiO_2_ NPs, as sonosensitizers, possess a low quantum yield of ROS due to rapid electron (e^-^)-hole (h^+^) recombination (50 ± 30 ns). Therefore, ultrafine titanium oxide nanorods (TiO_1+x_ NRs) modified with PEG were successfully prepared 117. Different from the oxygen-deficient structure of TiO_1+x_, which limited the US-triggered electron-hole pair recombination, the obtained PEG-TiO_1+x_ NRs showed high efficiency of US-triggered ROS generation. Moreover, TiO_1+x_ NRs had horseradish-like peroxidase activity and could catalyze H_2_O_2_ to produce •OH for CDT. Therefore, under ultrasound therapy, PEG-TiO_1+x_-NRs can be used as a sonosensitizer and CDT agent for highly effective tumor ablation. However, direct delivery of sonosensitizers to tumor tissue is limited by inefficient delivery and potential toxicity. Here, Professor Chen reported a TME-responsive 2D nanosonosensitizer/nanocatalyst (Ti_3_C_2_/CuO_2_@BSA) that achieved high-performance synergistic CDT/SDT tumor treatment by generating sonosensitizers in situ 118. The integration of CuO_2_ nanoparticles on 2D Ti_3_C_2_ enabled in situ generation of H_2_O_2_ under acidic tumor conditions for oxidation of Ti_3_C_2_ to generate TiO_2_ nanosensitizers, while the carbon matrix enhanced the separation of e^-^ and h^+^ after oxidation, further improving the SDT effect. Furthermore, ultrasound irradiation also enhanced Cu^+^-induced Fenton-like responses to generate more ROS, thus synergizing SDT tumor treatment. The experimental results proved the synergistic therapeutic effect of SDT/CDT *in vitro* and *in vivo* (**Figure 4C**). In addition, piezoelectric semiconductors were used to improve SDT and increase ROS generation activity through US-induced carrier separation and band bending. Benefiting from the piezoelectric effect, the piezoelectric heterostructure of Cu_2-x_O-BaTiO_3_ was prepared as a sonosensitizer and chemodynamic agent to improve the generation of ROS and the synergistic CDT/SDT treatment effect on refractory breast cancer in mice 119.

Au NPs is an another sonosensitizer candidate which has been reported for synergetic CDT/SDT 120. A novel US and GSH bis-responsive JNP vesicles (JNP-Ve) sonosensitizer that was prepared by grafting the block copolymer with hydrophilic thioglycol (PEG-SH) block and hydrophobic ROS-sensitive poly-(1,4-phenylacetone-dimethylthione) (PPADT-SH) block onto Janus Au-MnO NPs 121. Subsequently, under the stimulation and initiation of US, vesicles were broken down into small Janus Au-MnO NPs). And then GSH initiated MnO degradation, releasing Mn^2+^ for CDT, while releasing a smaller size Au NP with many cavitation nucleation sites for SDT. This two-step dissociation strategy not only improved the penetration of NP, but also increased the generation of ROS due to cavitation and Mn^2+^-induced Fenton-like reaction and achieved synergistic SDT/CDT to inhibit in situ liver tumor growth (**Figure 4D**).

In conclusion, CDT/SDT combination therapy can significantly improve the treatment efficiency of tumors by increasing total ROS production. Hypoxia may lead to severe resistance to O_2_-dependent SDT, which could lead to failure of treatment. CDT relies on endogenous rather than external energy stimulation. However, tumor endogenous response substrates are limited, which limits the therapeutic effect of CDT. When CDT is combined with SDT, the enlarged production of ROS can significantly improve the therapeutic effect on tumors. Organic sonosensitizers are loaded in well-stabilized carriers to avoid premature exposure and transport to the tumor site with minimal loss. The high tumor specificity of inorganic sonosensitizers integrating CDT and SDT allows them to better accumulate in tumor cells. However, compared to PTT and PDT, the mechanism of SDT is more complex. At present, there are many studies on the mechanism of SDT, but there is still no clear conclusion, and more research is needed on the synergy of multiple mechanisms leading to cell death in the future.

### CDT in Combination with RT

RT is the main approach for clinical cancer treatment. It is a kind of local treatment method that uses radiation to treat tumors. ROS-mediated DNA damage is the main mechanism of radiation-induced cell death 122. However, due to the radiation resistance of tumor cells, the therapeutic effect of RT is unsatisfactory 123. Tumor hypoxia is a major cause of RT resistance 124. Combining RT with CDT is a promising strategy to improve the efficacy of tumors. On the one hand, Fenton and Fenton-like reagents can catalyze H_2_O_2_ to produce O_2_ to alleviate tumor hypoxia for enhanced RT, and X-ray irradiation can enhance mitochondrial respiration and increase H_2_O_2_ production for enhanced CDT. On the other hand, CDT produces •OH in situ can significantly increase oxidative stress, enhance the radio-sensitivity of tumor cells, further improve the anti-tumor effect of RT.

**Figure 5:**
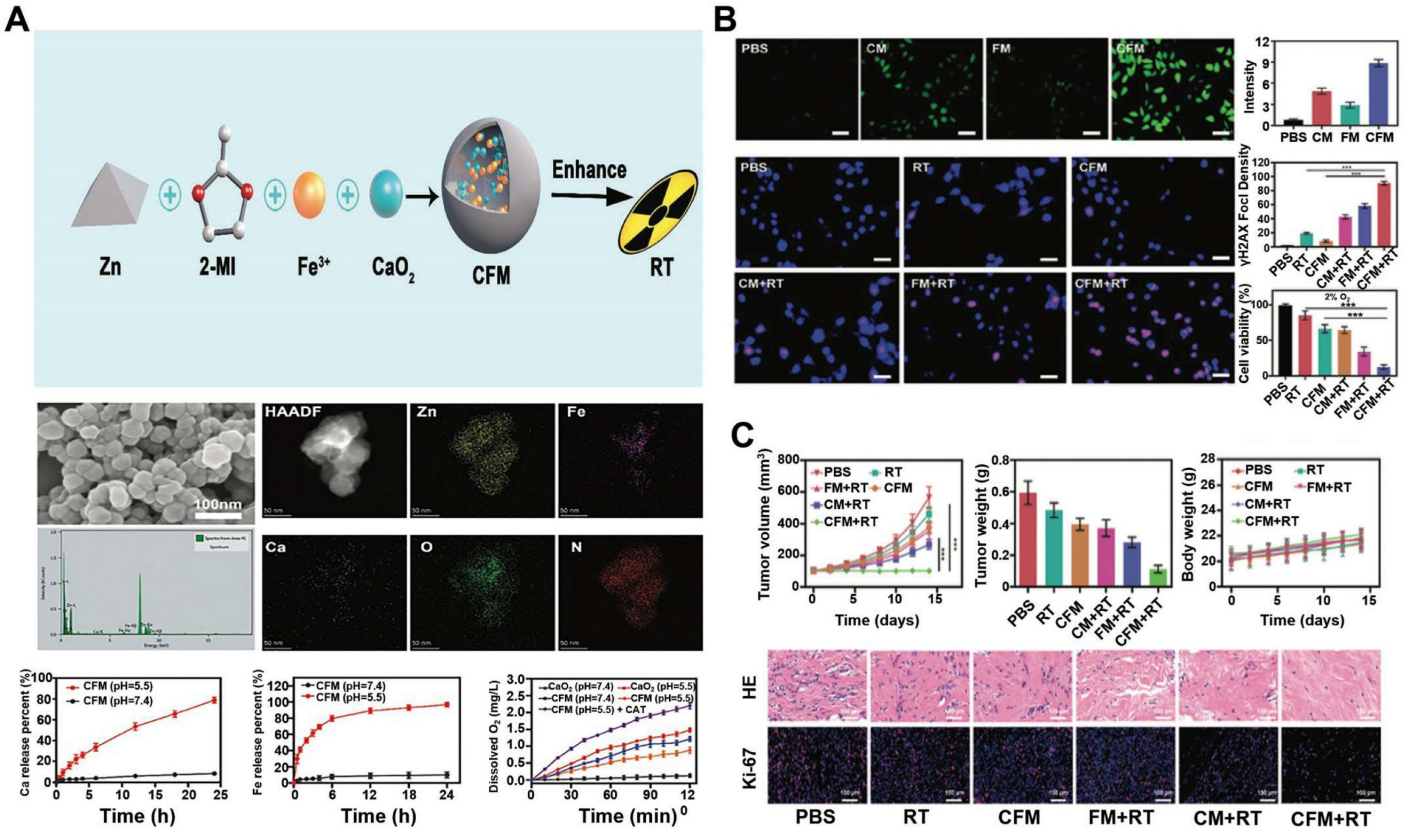
(A) Schematic illustration of CFM supplying intratumoral H_2_O_2_ and O_2_ for enhanced chemodynamic therapy and radiotherapy. SEM images of CFM; EDS mapping of CFM after washing with distilled water; Ca^2+^ and Fe^3+^ release in buffer solutions with different pH values; oxygen generation under a range of experimental conditions was evaluated using a dissolved oxygen meter. (B) Assessment of the *in vitro* efficacy of CFM. DCFH-DA was visualized and quantified in tumor cells treated as indicated. Scale bars = 20 μm. Representative images of DNA fragmentation and nuclear condensation within tumor cells following the indicated treatments, with DAPI and g-H2AX being used to visualize nuclei and DNA fragmentation, respectively. Scale bars: 50 μm. Quantitative assessment of γ-H2AX foci density (γ-H2AX foci/100 /μm^2^) for >100 cells per treatment condition. Following control, RT, CFM, FM + RT, CM + RT, and CFM + RT treatment, an MTT assay was used to assess 4T1 tumor cell survival under 2% O_2_ conditions (n = 5). *P < 0.05, **P < 0.01, ***P < 0.005; Student's t-test. (C) Tumor volume and body weight curves in mice bearing Representative H&E and Ki-67 stained sections from mice treated as indicated. Data are means ± SD (n = 5). *P < 0.01, **P < 0.005, ***P < 0.001; Student's t-test. Reproduced with permission 125. Copyright 2020, The Royal Society of Chemistry.

Nanoscale metal-organic frameworks (nMOFs) pave the way for enhanced radiation energy deposition at tumor sites. In a study, a nanoplatform with MOF structure (called CFM) formed by the co-doping of CaO_2_ nanodots and Fe^3+^ ions were developed 125. As an oxidative stress amplifier, CFM could achieve self-sufficient H_2_O_2_ and O_2_ for enhanced CDT and RT. CFM degraded rapidly at a lower pH and hypoxic TME to release Fe^3+^, Ca^2+^, O_2_ and H_2_O_2_. Fe^3+^ was subsequently reduced to Fe^2+^ by GSH, which could participate in the Fenton reaction and induce apoptosis. The production of O_2_ has also been confirmed to overcome the RT resistance (**Figure 5A**). CFM-mediated *in vitro* toxicity was indirectly demonstrated by DCFH-DA staining and γ-H2AX immunofluorescence staining. As shown in** Figure 5B**, the fluorescence intensity of the CFM treatment group increased significantly. Due to the presence of CaO_2_, CFM could considerably increase the intracellular H_2_O_2_ level and consume GSH. The resulted H_2_O_2_ was further catalyzed by the Fenton-like reaction with Fe^3+^ ions to generate •OH. Moreover, results from the γ-H2AX immunofluorescence staining showed that the double-stranded DNA broke within patient-derived xenograft (PDX) tumor cells for the CFM + RT treatment group, indicating that CFM enhanced the radiation sensitivity of tumor cells as well as intracellular oxidative stress. MTT results proved that the cell viability in the CFM + RT combined treatment group was significantly decreased. Next, we evaluated the *in vivo* anti-tumor efficacy of CFM in mice with PDX tumors of loaded bladder cancer. FM + RT and CM + RT treatment can achieve partial inhibition of tumor growth compared to the control group of animals with rapid tumor growth. In contrast, the combination of CFM + RT treatment can obtain the most robust tumor growth inhibition. Ki-67 staining indicated that the combination of RT + CFM treatment significantly reduced the number of cancer cells, and mice's weight monitoring and H&E staining demonstrated the system safety of CFM nanocomposites (**Figure 5C**).

High-Z metal nanoparticles include bismuth, hafnium, gold, gadolinium, silver and metal oxide nanoparticles are widely used for enhanced RT/CDT combination therapy 126, 127. Li's group developed a Cu^2+^ doped BiOCl nanotherapeutic platform called BCHN (BiOCl/Cu^2+^-H_2_O_2_ @PVP) 128. BCHN could release self-carried H_2_O_2_ in a slightly acidic environment. Then, BCN interacted with excessive GSH in tumor cells to consume GSH. At the same time, Cu^2+^ was reduced to Cu^+^ by GSH, Cu^2+^ and Cu^+^ interacted with self-sufficiency and endogenous H_2_O_2_ to produce large amounts of •OH for CDT and O_2_ for alleviating tumor hypoxia. The sustained production of •OH and depletion of GSH regulated intracellular oxidative stress levels, achieving oxidative stress-enhanced CDT and RT. Besides, the presence of high Z element bismuth could also help locally accumulate more radiation power at the tumor site, which would no doubt enhance radiotherapy. Synergistic CDT/RT effectively inhibited tumor cell proliferation and improved tumor control.

In addition, Hafnium-based metal-organic frameworks (Hf-nMOFs), formed from a mixture of Hf^4+^ ions and organic bridging ligands, have also emerged as radiation sensitizers due to their excellent X-ray energy absorption and conversion capabilities. The porous and tunable morphology can so far be loaded with different components for a variety of applications 129, 130. With this in mind, introducing Fe^3+^ into Hf-nMOFs (named Hf-BPY-Fe) could make the platform suitable for CDT and RT 131. Hf-BPY-Fe has been shown to improve the efficacy of radiation therapy through a combination of mechanisms: the sustained ROS induced by the Fenton reaction altered the cell cycle distribution and contributed to the increased radiosensitivity of tumors. Simultaneously, after irradiation, Hf^4+^ in Hf-BPY Fe could generate a large number of high-energy electrons while relaxing into a low-energy state in the nMOF pores, creating an electron-rich environment. These enriched electrons in nMOF accelerated the reduction of Fe^3+^ to Fe^2+^, further promoted the generation of •OH in the Fenton process and achieved synergistic CDT and RT tumor therapy. Moreover, Hf-BPY-Fe could delay the DNA damage response by interfering with certain proteins involved in the DNA repair signaling pathway. The system offered a new approach for radiosensitization in the whole process of tumor treatment and intervention of eventual DNA repair (**Figure 5D**).

Furthermore, metal oxides-based nanozymes have been rapidly developed in CDT/RT in recent years 132,133. In one study, the researchers chose the classical Fe_3_O_4_ NPs as a template to design a nanozyme consisting of SnS_2_ nanoplates and Fe_3_O_4_ quantum dot with moving or mixed redox states (Fe^2+^/Fe^3+^) (named SnS_2_@Fe_3_O_4_ NPs, SF NPs) 134. In the presence of X-rays, the redox cycle of these nanomaterials with mobile or mixed redox states was accelerated to enhance their enzymatic activity. The SF NPs nanozymes with peroxidase-like activity could decompose H_2_O_2_ into highly toxic ROS. The SnS_2_ cofactor, which acted as an electron donor, could be triggered to transfer electrons to Fe_3_O_4_ under external X-ray irradiation so as to promote the regeneration of Fe^2+^ on the surface of Fe_3_O_4_. The regenerated Fe^2+^ ions provided a steady stream of raw materials for the Fenton reaction, then reacted with H_2_O_2_ to continuously generate ROS, leading to severe tumor damage. Since the SF NPs selectively exerted enzyme activity in TME under X-ray irradiation, it could help reduce the damage to normal tissues. This nanocomposite provided a new avenue for developing synergistic effects of radiotherapy and CDT based on X-ray-enhanced enzyme effects. Another study reported a novel X-ray responsive Cu_2_(OH)PO_4_ NPs that could simultaneously respond to H_2_O_2_ and X-ray for CDT/RT 135. Under X-ray irradiation, the NPs generated Cu^I^ sites through photoelectron transfer that were used as Fenton-like catalysts to break down overexpressed H_2_O_2_ into •OH. Therefore, X-ray-triggered Fenton-like reaction provides a more controllable and reliable method to improve CDT/RT and reduce side effects. This strategy confirms that the radiosensitization process can only be carried out in hypoxic tumors, minimizing damage to surrounding healthy tissue. Additionally, in the latest study, Prof. Sun's team achieved MR-guided synergistic CDT/RT using only superparamagnetic iron oxide nanoclusters (SPIONC) 136. After pulmonary delivery in situ lung cancer, SPIONC had an enhanced penetration and retention effect in lung cancer, then broke down into smaller nanoparticles in an acidic microenvironment to improve tissue penetration and released more iron ions for CDT. The composition of this system is very simple, but the fate of nanoclusters remains to be studied. Besides, in-depth studies of the long-term toxicity and metabolism of clusters are needed.

In summary, the high radiation dose required in the RT can have huge side effects on healthy tissues, and the mild doses of X-ray can overcome the above shortcomings. In addition, mild X-ray doses can activate the surface properties of nanomaterials and improve Fenton catalysis, X-ray irradiation can also enhance mitochondrial respiration and increase the production of H_2_O_2_. In short, the combination of RT and CDT show the respective advantages of X-ray high tissue penetration and in situ ROS generation for intratumor-Fenton/Fenton-like reactions to achieve the synergistic increase of ROS levels. However, the efficacy of ROS-mediated tumor therapy is severely limited by tumor hypoxia and ROS-induced protective autophagy. Reactive nitrogen species (RNS)-activated nitrosative stress for tumor treatment is expected to overcome many drawbacks of traditional ROS therapy and significantly improve the therapeutic effect of hypoxic tumors. Intracellular nitrosative stress induces intracellular biomacromolecular damage mainly through peroxynitrite (ONOO^-^), nitrite, hydrogen peroxide, or peroxidase pathways, resulting in a series of cell death. Therefore, the overproduction of ONOO^-^ in tumors is expected to be an effective method for RT sensitization. For example, Yang reported the controllable ONOO^-^-enhanced silica nanoscintillator (PEG/SCNPs@DMSN-SNO-g-C_3_N_4_, P/SMNO-C) as a promising strategy to enhance intracellular nitrosative stress, making it an effective X-ray-controlled RT treatment for postoperative colon cancer 137. Recently, two-dimensional nanoplatforms containing NO donor-modified LiYF4: Ce scintillators and graphite carbonitride nanosheets have also been developed for on-demand generation of highly cytotoxic ONOO^-^, further improving the efficacy of radiotherapy by directly inducing mitochondrial and DNA damage 138. Moreover, Yang's group also developed a versatile core-shell radiosensitizer (SCNPs@DMSN@CeOx-PEG) for image-guided X-PDT by optimizing energy matching between the nanooscillators core and the semiconductor shell 139. Therefore, CDT/RT based RNS has great perspectives for improvement in the future.

### CDT in Combination with MHT

MHT was first proposed by Gilchrist in 1957 140. MHT relies on the heat dissipation of magnetic NPs in an alternating magnetic field (AMF) to heat diseased tissue and damage cancer cells. MHT is a non-invasive in situ treatment with no depth limitation and minimal adverse effects on surrounding healthy tissue 141, 142. However, the efficacy of MHT is limited by tumor recurrence due to upregulation of heat shock proteins (HSPs) and thermal resistance due to overdose administration of magnetic NPs 143, 144. ROS and competitive adenosine triphosphate (ATP) depletion are essential targets that can block excessive HSP production 145. What's more, the high temperatures generated by magnetic NPs can accelerate the Fenton reaction. As a result, the combination of CDT and MHT is expected to improve therapeutic outcomes.

**Figure 6:**
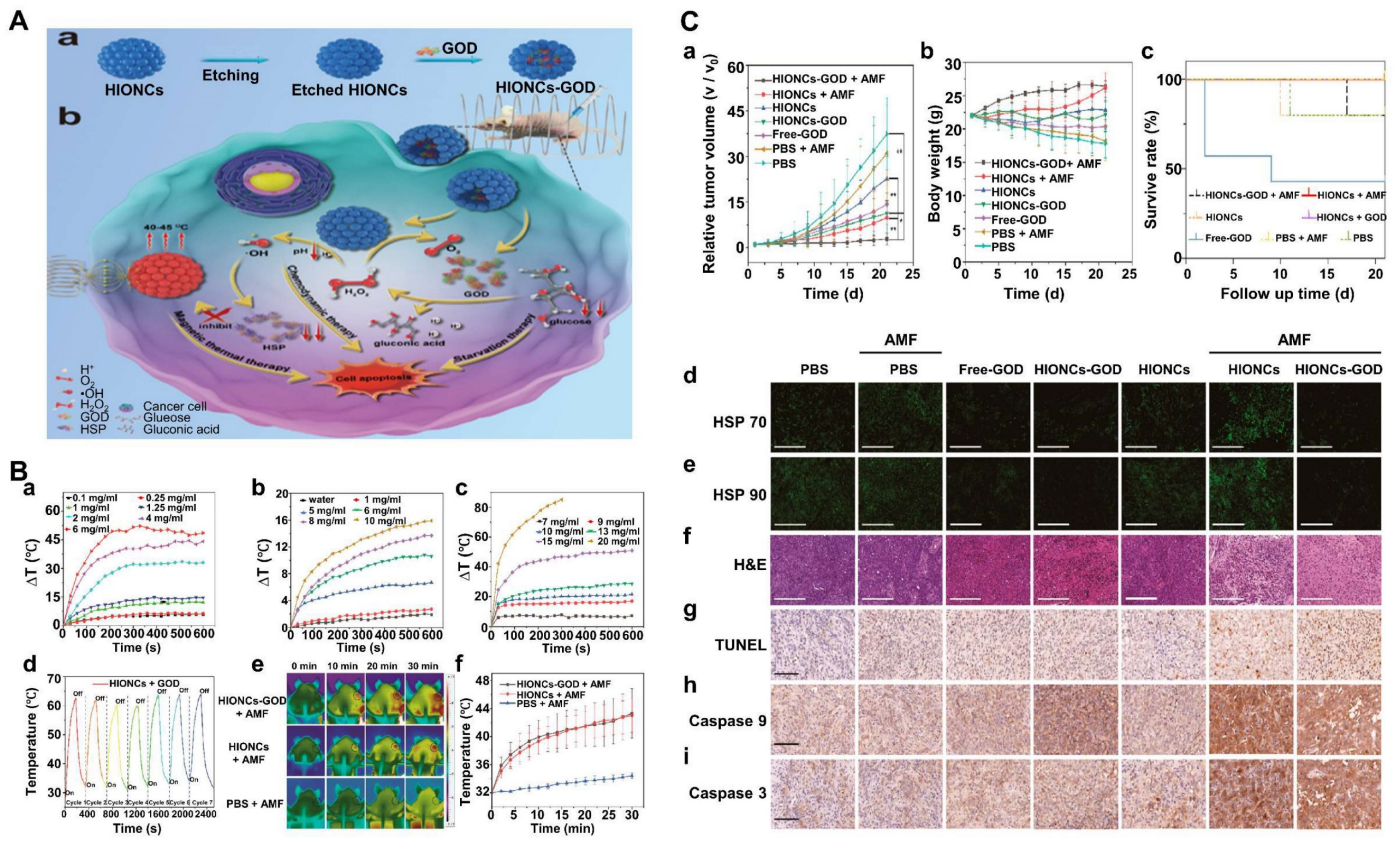
(A) a) Synthesis of etched HIONCs and the encapsulation of GOD into HIONCs. b) GOD-induced starvation chemodynamic therapy for enhanced magnetic hyperthermia therapy in the hypoxic tumor microenvironment. HIONC-mediated oxygen replenishment simultaneously enhanced starvation therapy. (B) Quantitative temperature curve of ultrapure water and HIONCs *in vitro*: a) microcentrifuge tube, b) gel, and c) cattle liver. d) Heating curves of the HIONCs-GOD dispersion in water (10 mg/mL) exposed to the AMF of 513 kHz at 1001.1 A/m for seven on/off cycles. e) Thermal images of PC3 tumor-bearing mice after exposure to the AC magnetic field, including HIONCs-GOD + AMF, HIONCs + AMF, and PBS + AMF. (f) Temperature curve against time for the HIONCs-GOD, HIONC, and phosphate-buffered saline (PBS) groups *in vivo*. The temperature changes were recorded with a thermal infrared camera. (C) (a) Relative tumor volume after treatment. The animals were monitored every other day (n = 5, *P < 0.05, **P < 0.01). (b) Relative body weight of mice in the different groups after treatment. (c) Survival curves of different groups during the 21 d observation period. Representative images of PC3 tumor tissue sections at 24 h after treatment with PBS, PBS + AMF, free GOD, HIONCs-GOD, HIONCs, HIONCs + AMF, or HIONCs-GOD + AMF: immunofluorescence staining with (d) anti-HSP70 and (e) anti-HSP90 antibody, (f) H&E, and (g-i) immunohistochemical staining. Green: HSP70 and HSP90 expressing region. The white scale bar in (d-f) is 200 μm. (g) TUNEL, (h) caspase 9, (i) caspase 3. The black scale bar in (g-i) is 100 μm. Reproduced with permission 148. Copyright 2020, American Chemical Society.

Iron oxide, manganese ferrite, and zinc ferrite are used as magnetic NPs, which can also be used as MRI contrast agents for disease diagnosis and are therefore particularly suitable for in situ CDT/MHT of cancers 146, 147. A recent study showed that a hollow iron oxide nanocatalysts loaded with GOD (named HIONCs-GOD) were successfully fabricated for chemodynamic-hyperthermia synergistic therapy 148. Fe^2+^ mediated the Fenton reaction to generate ROS, which induced cell apoptosis and down-regulated HSP expression, thereby alleviating heat resistance. On the other hand, Fenton efficiency was also enhanced by HIONCs-mediated MHT, GOD-mediated H_2_O_2_ accumulation, and elevated acidity of the tumor microenvironment. In addition, the peroxidase-like activity of HIONCs facilitated the conversion of H_2_O_2_ to O_2_, overcoming the limitation of tumor hypoxia on therapeutic efficacy (**Figure 6A**). When being exposed to an AMF for 10 min, the temperature increased to 50°C at a concentration of 6 mg/mL of HIOCs. While a low concentration of HIOCS, saying 0.1 mg/mL, only led to a slight temperature increase by about 3°C, suggesting that the rate of temperature increase was related to the concentration of HIONCs. And the results of the temperature curve showed that HIOCS-GOD was favorable to magnetic therapy, and the loading of GOD did not affect its magnetic-thermal performance. The magnetothermal effect of HIONCs-GOD on PC3 tumor-bearing mice showed the highest tumor temperature in the HIONCs + AMF group and the HEIONC-GOD + AMF group rose rapidly to nearly 43°C, compared to only a slight increase in body temperature in the PBS + AMF group (**Figure 6B, a-f**). The therapeutic effect of the nanocatalysts *in vivo* further suggested that nude mice in the HIONCs-GOD + AMF group showed more pronounced tumor growth inhibition, good weight gain and better survival rates than other groups, which was attributed to the synergistic combination of CDT, MHT, and starvation therapy and the superior biocompatibility of HIONCs-GOD (**Figure 6C, a-c**). Furthermore, tumor proliferation, necrosis testing, and H&E staining results showed HIONCs-GOD + AMF-treated tumor cells underwent severe apoptosis and morphological changes. More importantly, the expression levels of HSP70 and HSP90 in the HIONCs-GOD + AMF group were significantly lower than those in the other groups due to the HIONCs-GOD induced starvation effect and the production of large amounts of H_2_O_2_ by GOD (**Figure 6C, d-i**). This multifunctional nanocarrier system achieved synergistic CDT/MHT/starvation treatment of prostate tumors.

To improve the peroxidase-like activity and magneto-thermal conversion efficiency of Fe_3_O_4_ nanoparticles, hollow Fe_3_O_4_ mesocrystals (MCs) were successfully constructed by an improved solvothermal method. The high-efficiency MH-CDT synergistic tumor treatment using a single Fe_3_O_4_ component was realized. Due to the unique magnetic properties of the mesocrystal structure, the Fe_3_O_4_ MCs exhibited an excellent magneto-thermal conversion efficiency and higher peroxidase-like activity than Fe_3_O_4_ polycrystals (PCs) 149. The Fe_3_O_4_ MCs-mediated Fenton reaction induced the decomposition of H_2_O_2_ to •OH, which damaged tumor cells and reduced the expression of HSPs, making tumor cells more susceptible to MH attack. Besides, MH-mediated hyperthermia in situ could in turn promote CDT and achieve a mutually reinforcing effect between MH and CDT, effectively eradicating tumors.

In another recent study, a mitochondria-targeted magnetothermal nanozyme (Ir@MnFe_2_O_4_NPs) was also used for efficient CDT/MHT cancer therapy 150. Mitochondria is an essential organelle responsible for cellular energy production and cell survival, and they are also involved in many critical physiological processes, such as apoptosis, signal transduction, and ROS production. The above properties make mitochondria excellent targets for tumor therapy 151, 152. Based on the above system, MnFe_2_O_4_ NPs had a good magnetic caloric performance and had an excellent catalytic activity. After modification by cyclometallated iridium (III) complexes, the excellent mitochondria-targeting agent, MnFe_2_O_4_ was delivered to the mitochondria for precise and effective cancer treatment. The superior magneto-thermal properties of Ir@MnFe_2_O_4_ NPs caused local temperature increases, resulting in mitochondrial damage and cell death. In addition, Fe (III) in MnFe_2_O_4_ NPs was reduced to Fe (II) by GSH, which lead to GSH depletion, and the increase of intracellular Fe (II) also enhanced the rate of the Fenton reaction, producing more •OH. Furthermore, disruption of intracellular redox homeostasis further amplified the effect of MHT. Moreover, Ir@MnFe_2_O_4_NPs could be used for two-photon imaging and MRI, allowing for more precise and effective cancer treatment.

Therefore, combined CDT and MHT are considered to be an ideal strategy for synergistic treatment of cancer. This mutually reinforcing nanosystem has been shown to overcome the shortcomings of both MHT and CDT. The generation of ROS during CDT can inhibit the expression of HSPs and reduce the thermal tolerance of cells, thereby significantly improving the therapeutic efficiency of MHT. The increase in temperature caused by MHT can speed up the Fenton reaction and improve the catalytic efficiency of the material. Unlike PTT, which uses light to generate heat to kill tumors, MHT can handle deep tumors in various tissues due to its superior tissue penetration. However, it is worth noting that in order to achieve the ideal treatment temperature, the concentration of materials used in MHT is usually higher, which may increase the potential toxicity of the therapy. Therefore, there is an urgent need to develop materials with high magnetocaloric conversion capabilities.

### CDT in Combination with Immunotherapy

Immunotherapy shows an excellent potential in the treatment of malignant tumors by reactivating anti-tumor immune cells and overcoming immune evasion of tumors 153. Based on the mechanisms of immunotherapy, a variety of immunotherapeutic tools have been developed, including immune checkpoint inhibitors, small molecule immunomodulators, cancer vaccines, lysozyme viruses, engineered T cells, and agonistic antibodies against co-stimulatory receptors 154-156. Immune checkpoint blocking (ICB) therapy based on programmed cell death 1 (PD1) and programmed death ligand 1 (PDL1). Cytotoxic T-lymphocyte associated protein 4 (CTLA-4) is one of the effective strategies to activate antitumor immunity, which has made great progress in treating a wide range of malignancies in recent years 157. However, the effectiveness of immunotherapy is severely affected by the immunosuppression of microenvironment 158. In addition, TME also plays an important role in immunosuppression to aggravate cancer proliferation and metastasis. As a result, both tumor cells and associated TMEs lead to low response rates, ultimately leading to unsatisfactory immunotherapy. Elevated levels of tumor oxidative stress by CDT can induce immunogenic cell death (ICD) and reverse immunosuppression of TME, promoting an anti-tumor immune response. Based on this, combining CDT immunotherapy is considered ideal for improving tumor outcomes.

**Figure 7:**
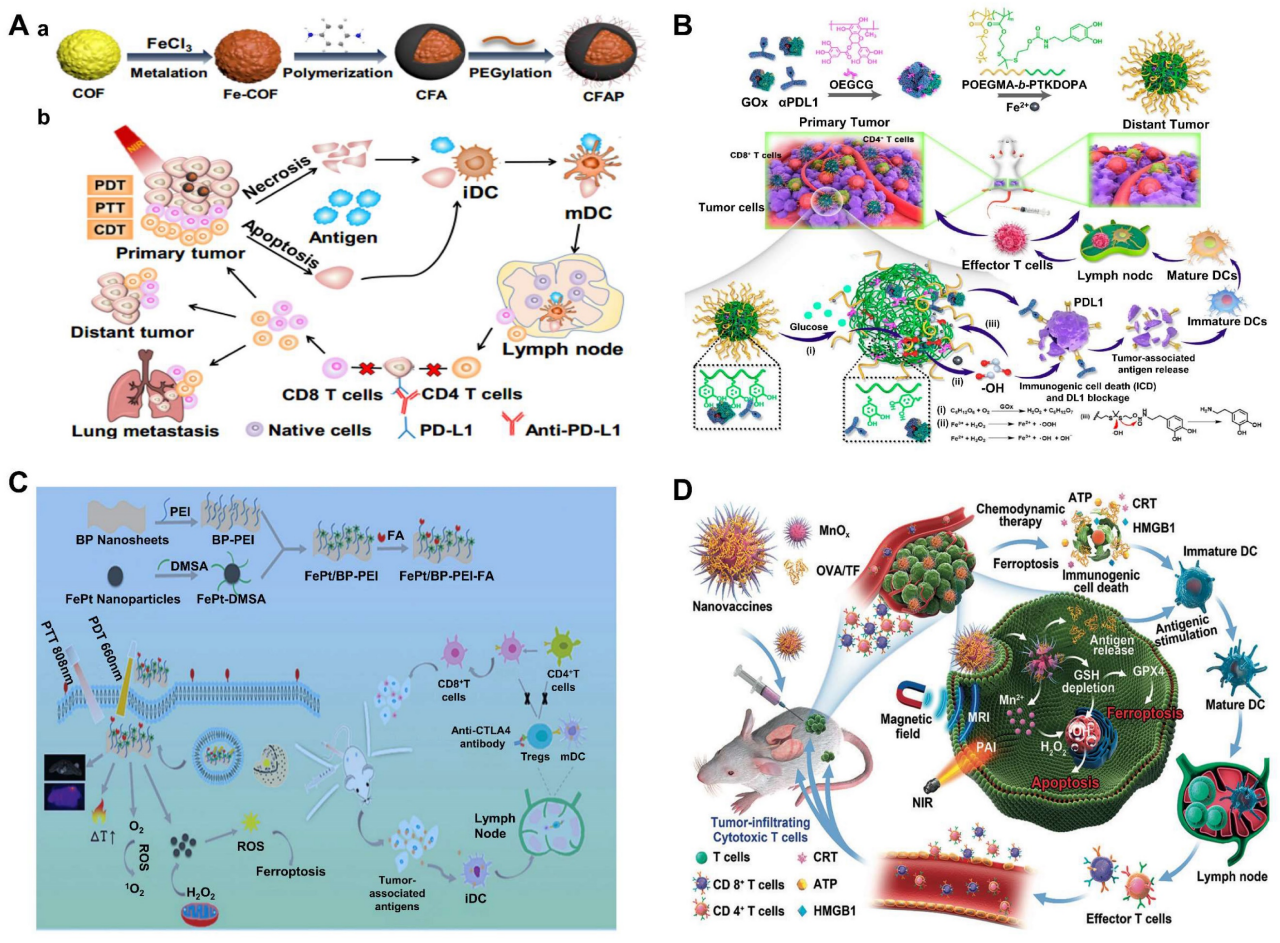
(A) Illustration of a) preparation and b) synergistic cancer therapy of CFAP. Reproduced with permission 159. Copyright 2020, American Chemical Society. (B) Schematic illustration for the a) preparation of P/G@EF-TKNPs and b) mechanism of *in vivo* CombinationTherapy of CDT and ICB 161. Copyright 2022, American Chemical Society. (C) Schematic illustration of the preparation of FePt/BP-PEI-FA NCs and dual-modal imaging (MR and thermal imaging)-guided synergistic PTT/PDT/CDT cancer therapies as well as photothermally-enhanced immunotherapy. Reproduced with permission 163. Copyright 2020, The Royal Society of Chemistry. (D) Illustration of MnOx-OVA/tumor cell fragment (TF) nanovaccines for MR/PA dual-mode imaging-induced cancer immunotherapy. Reproduced with permission 168. Copyright 2020, Wiley-VCH.

Rationally designed CDT nanosystems combined with ICB therapy show great potential to maximize the anticancer efficacy. Herein, a multifunctional covalent organic framework (COF)-based nanocomposite was designed for synergistic photo-, chemodynamic- and immunotherapies 159. Specifically, 1,3,5-tris(4-aminophenyl) benzene (TAPB) 2,5-dimethox-yterephthaldehyde (DMTP)-COF was synthesized at room temperature, followed by the introduction of iron trichloride to form Fe-COF, then poly (p-phenylenediamine) was polymerized on the surface of the COF using Fe^3+^ as the oxidizing agent. Fe^3+^/Fe^2+^ redox couples will further catalyze the overexpressed H2O2 in the tumor to •OH by the Fenton reaction, and the increased temperature induced by poly(p-phenylenediamine) also accelerated the production of •OH and enhanced CDT. Furthermore, in the presence of anti-PDL1 checkpoint blockade therapy, tumor-associated antigens could induce a robust anti-tumor immune response and effectively inhibit tumor metastasis (**Figure 7A**). For targeted delivery anti-mouse programmed death ligand 1 antibody (αPDL1) and enhanced immune efficiency, Ge et al. demonstrated a ROS-responsive αPDL1/GOx nanocomplex for the combination of CDT and ICB. Nanocomplexes (P/G@EF-TKNPs) were prepared by complexing proteins, αPDL1/GOx, and oligomerized (-)-epigallocatechin-3-O-gallate (OEGCG), then chelating them with Fe^3+^, and finally overlaying the block copolymer into POEGMA-b-PTKDOPA (**Figure 7B**). OEGCG, as a promising natural protein carrier adjuvant, has been demonstrated excellent performance in αPDL1 delivery 160. POEGMA blocks acted as shells that protect the integrity of nanocomplexes in blood circulation, and PTKDOPA fragments as chelating agents for the formation of Fe^3+^ and protein complex. When P/G@EF-TKNPs entered tumor cells, GOx catalyzed glucose to produce H_2_O_2_ for enhanced CDT and promoted the release of tumor-associated antigens (TAAs). Moreover, the released αPDL1 bound specifically to PDL1 on tumor cells for ICB. Finally, the nanocomplexes induced superior systemic immune responses, effectively inhibiting the growth of primary and distant tumors in the mice bilateral 4T1 tumor models 161.

CTLA-4 blockers capable of altering the immunosuppressive microenvironment were also used to treat metastatic tumors 162. In a study, FePt NPs were loaded onto PEI-modified black phosphorus nanosheets (BPNs) and then modified with folic acid to achieve the FePt/BP-PEI-FA NCs 163, which exhibited excellent Fenton reaction for CDT, photothermal and photodynamic effects under 808 nm for PTT and 660 nm NIR irradiation for PDT, respectively. FePt NPs, as a ferroptosis agent, could convert endogenous H_2_O_2_ to ROS through the Fenton reaction. The synergistic effect of PTT, PDT and CDT effectively inhibit tumor growth. Meanwhile, tumor-associated antigens released during PTT treatment stimulated anti-tumor immune responses. By further combining with CTLA-4 blocking therapy, it efficiently controlled residual and metastatic tumor growth (**Figure 7C**). In addition, bacteria can activate innate and adaptive immune responses *in vivo*, and using microbes is an ideal strategy to combine ICB with CDT in precision ROS therapy against tumors. For example, Au@Pt core-shell nanozyme decorated on the surface of bacteria could be promising agents of CDT in cancer treatment and the further immunotherapeutic effect was induced by bacterium-induced 164. In another study, Fc-based MOF Vk3-loaded cascading catalytic nanoplatform was reported for enhanced CDT/immunotherapy based on Vk3-induced H_2_O_2_ amplification and •OH-mediated ICD for immunotherapeutic effect 165.

To reverse the immunosuppressed tumor microenvironment and acquire an immune memory effect, Liu and his colleagues reported a novel artificial enzyme Cu_2-x_Te-NEs with tunable enzyme-mimetic activity under NIR-II light. The Cu_2-x_Te-NEs had both glutathione oxidase and peroxidase-like activities, which could efficiently catalyzed GSH and H_2_O_2_, producing large amounts of ROS 166. Besides, the defect-induced NIR-II absorption endowed Cu_2-x_Te NEs with NIR-II photothermal effect, and the enzymatic activity and Fenton reaction rate of Cu_2-x_Te-NEs were significantly enhanced under NIR-II light. Moreover, the sustained oxidative stress generated by the Cu_2-x_Te-NEs reversed the immunosuppressed tumor microenvironment and established an immune memory effect that eradicated primary and metastatic tumors and inhibited tumor recurrence. Additionally, by optimizing composition and morphology, a unique eggshell nanohybrid (Fe_3_O_4_@C/MnO_2_-PGEA, FCMP) with innate immunomodulatory effects was constructed. The intrinsic immunomodulatory effects of FCMP had been used to reprogram macrophages to the M1 phenotype and induce maturation of dendritic cells, and the fully exposed Fe_3_O_4_ nuclei and MnO_2_ shells could maximize CDT and significantly inhibit the growth of primary and distant tumors 167.

Furthermore, nanovaccine-based immunotherapies are known for their high efficiency, minimal side effects and toxicity, antigen-specific immunity, and long-term immunological memory effects. As an integral component of cancer nanovaccines, immune adjuvants play a vital role in antigen delivery and immune enhancement. In one study, MnOx nanospikes (NSs) with large mesoporous structures were evaluated as a TME-responsive nano-adjuvants. They had extremely high loading efficiency with ovalbumin (OVA) and tumor cell debris. MnOx immune adjuvants were proven to induce ICD via CDT and ferroptosis 168. Using OVA protein as a model antigen, the MnOx-OVA-treated group exhibited the best maturation of dendritic cells (DCs) and T-cell activation and the highest levels of interleukin-6 (IL-6) and tumor necrosis factor-α secretion. 4T1 tumor cell fragments (TF) were used as cognate antigens for the treatment of 4T1 tumors *in vivo*. Attributable to the synergistic effect of MnOx-mediated ICD induced by CDT and TF-mediated immunotherapy, effective primary and distal tumor growth inhibition was observed in the treatment group (**Figure 7D**). In another recent study, MoS_2_-CuO heterogeneous nanocomposites were first designed by integrating semiconducting CuO and flower-like MoS_2_ in a two-step hydrothermal method, then loaded with both BSA and the immune adjuvant imiquimod (R837). The obtained MoS_2_-CuO@BSA/R837 (MCBR) nanoplatform significantly achieved enhanced antitumor effects of synergistic CDT/immunotherapy/PTT and excellent CT/NIR/MRI multimodal imaging performance 169. In this nanoplatform, CuO was a typical CDT reagent that reacted with the overexpressed H_2_O_2_ in TME to generate •OH through a Fenton-like reaction, and the photothermal effect of MoS_2_ significantly increased the Fenton reaction rate and increased the •OH yield. At the same time, MCBR-based PTT and PT-enhanced CDT released tumor associated antigens (TAAs), which could bind to R837, an immunostimulatory adjuvant, that effectively inhibits primary and metastatic tumors by promoting DC maturation, secreting cytokines, and increasing CD4^+^ T cells and CDT cells.

Therefore, CDT combined immunotherapy has broad prospects in eradicating primary tumors, inhibiting metastatic tumors and preventing tumor recurrence. CDT can induce ICD and reverse immunosuppression of TME. However, CDT alone is often insufficient to reverse the immunosuppressive microenvironment. Thus, phototherapy is generally introduced in conjunction with CDT to boost immunity. Moreover, the combination of CDT and immunotherapy is still in its early stages, and their synergistic mechanisms still need further study due to complex immune mechanisms. In addition, it should be noted that excessive immune activation may also lead to an autoimmune response, which can induce autoimmune diseases. In summary, an in-depth exploration of the therapeutic mechanism and optimal regimen of CDT combined immunotherapy is the focus of current research.

### CDT in Combination with ST

Starvation treatment, by means of consuming nutrients in tumor cells, has been widely explored in tumor treatment 170. GOD/GOx is considered a key enzyme in the starvation therapy, which can effectively catalyze the oxidation of glucose to gluconic acid and H_2_O_2_, thereby cutting off the energy supply of cancer cells to inhibit their growth. This process is known as GOD-based starvation therapy. Nevertheless, due to the continuous consumption of O_2_ molecules and nutrients, as well as the spread and degradation of GOD in the tissue, it is difficult to eliminate tumors by relying on GOD alone. Hence, combined starvation therapy and CDT mediated by multifunctional nanomaterials based on GOD and Fenton reagent (Fenton-like reagent) have been proven effective in enhancing tumor therapy. GOD catalyzes glucose oxidation to gluconic acid and H_2_O_2_, resulting in reduced pH value and elevated H_2_O_2_ concentration, which can accelerate the efficiency of the Fenton reaction. In one study, GOD was loaded onto the surface of Cu^2+^-doped hollow mesoporous SiO_2_ nanoparticles (HMSN-Cu-GOD) for tumor-selective combination CDT and starvation therapy 171. After being internalized by tumor cells, the HMSN-Cu-GOD catalyzed the conversion of glucose to H_2_O_2_ for the next Fenton reaction in the presence of oxygen. And the glucose acid induced pH reduction promoted the release of Cu^2+^, which was further reduced by GSH and participated in the Fenton-like reaction. *In vitro* and *in vivo* experiments demonstrated that HMSN-Cu-GOD had superior synergistic effects on CDT and starvation therapy. For enhancing drug accumulation and therapeutic efficacy in tumors, a study reported peanut-shaped multifunctional nanomedicines (CuS-PGH-NMs) by encapsulating CuS in polylysine-glucose oxidase-HA shells, CuS-PGH-NMs showed significant uptake by tumor cells due to EPR effects and CD44 receptor-mediated targeting effects. The nanocomposite would decompose in cells, and the released GOx consumed endogenous glucose for starvation treatment. The excessive H_2_O_2_ was then converted to •OH by Cu^+^-mediated Fenton-like reaction for CDT. Furthermore, excessive H_2_O_2_ in turn accelerated Cu^2+^/Cu^+^ conversion for enhanced CDT, thereby achieving effective tumor growth inhibition through synergistic starvation/CDT therapy 172. To achieve long-term tumor retention of GOx, *in situ* gelatinized nanomedicine was prepared by mixing the hydrogel precursors N, N-dimethylacrylamide and polyethylene glycol diacrylate with GOx and GA-Fe nanocomplexes, which allowed long-term tumor retention of GOx and GA-Fe and destroyed the breast cancer tumors effectively 173.

**Figure 8:**
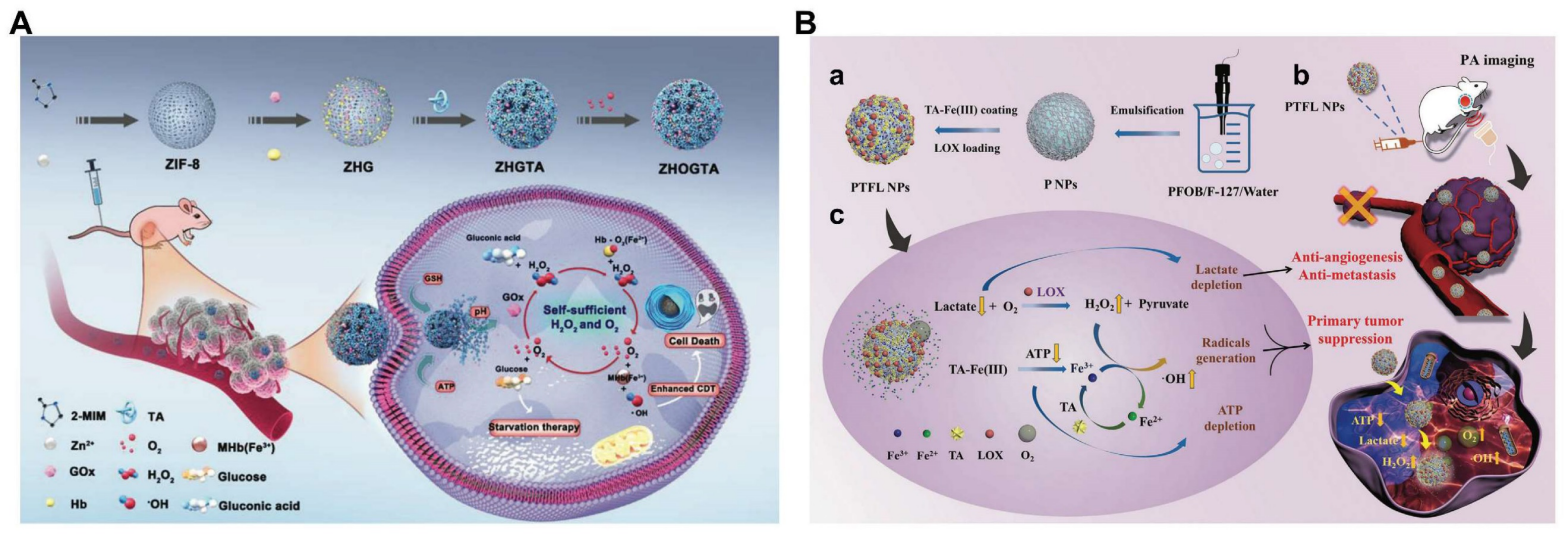
(A) Schematic illustration of the prepared process of nanocapsule and the strategies for synergistic therapy. The design of self-sufficient hydrogen peroxide and oxygen by using glucose oxidase and hemoglobin ultimately reinforced cascade reactions for tumor therapy. Reproduced with permission 176. Copyright 2022, Wiley-VCH. (B) a) Schematic illustration of PTFL NPs synthesis. b, c) Schematic illustration of PTFL NPs for PA imaging-guided synergistic and cascade metabolic-chemodynamic therapy. Reproduced under the terms of the http://creativecommons.org/licenses/by/4.0/ License 181, published by WILEY-VCH Verlag GmbH & Co. KGaA, Weinheim.

However, the glucose consumption efficiency of GOx is limited in the hypoxic TME. Although H_2_O_2_ can be converted to oxygen under the action of catalase, but the limited H_2_O_2_ in the tumor microenvironment also limits the intrinsic oxygen production. Based on the above situation, a great quantity of nanomedicines with self-supplied oxygen and hydrogen peroxide have been developed 174, 175. Besides, Professor Xu proposed a closed-loop strategy for controlled oxygen delivery, release and utilization 176. The study used ZIF-8 as a template to enrich proteins on the surface using its positive potential, hemoglobin (Hb) and GOx were then added, followed by adding tannic acid (TA) to construct the metal-protein-polyphenol network structure. Finally, oxygen was loaded onto the capsule by a strong affinity with Hb (**Figure 8A**). Hb is an excellent oxygen carrier and has peroxidase-like properties. After being internalized by cancer cells, GOx-mediated glycolysis continues to supply H_2_O_2_ for Hb catalysis, achieving enhanced ST and CDT. In this system, O_2_ can be released in time in two ways as needed: 1) depletion of O_2_ during glycolysis facilitated the release of O_2_ from Hb; 2) H_2_O_2_ can oxidize Hb to methemoglobin, promoting the release of O_2_ owing to the different affinity between oxygen and Hb/MHb. The nanomedicine demonstrated good GOx/Hb activity and ability to deliver GOx/Hb across membranes and was applied for the first time in the co-treatment of cancer at the small animal level.

The efficiency of GOx catalytic reactions is strongly dependent on oxygen concentration, but hypoxic conditions in TME result in low GOx activity. Thus, other types of starvation therapy have been explored. Amino acids are nutrients in cells and essential components of proteins, which play a fundamental role in maintaining the cytoskeleton and the cellular life cycle. The deficiency of amino acids may directly lead to the collapse of the cytoskeleton and disruption of cellular metabolism, resulting in cell death 177, 178. Therefore, the induction of amino acid depletion holds promise as an alternative approach to enhance tumor suppression. L-amino acid oxidase (AAO) catalyzes the production of amino acids α-ketoacids, releasing H_2_O_2_ and ammonia. α-ketoacids can regulate the pH of tumors, promote Fenton or Fenton-like reactions, and H_2_O_2_ can also continuously provide substrates for Fenton reactions. Based on this concept, Han's group used hollow Fe^3+^/ tannic acid (HFE-TA) nanocapsules as carriers to deliver AAO for synergistic tumor treatment 179. By wrapping it in the 4T1 cancer cell membrane, the biosafety of NPs was improved, and NPs were endowed with immune escape and tumor-targeting ability. AAO molecules specifically catalyzed the oxidative deamination of L-amino acids and lower pH at the tumor site, At the same time, the up-regulated H_2_O_2_ concentration promoted the HFe-TA-mediated Fenton reaction and enhanced •OH production. Benefiting from the functions mentioned above, this platform achieved synergistic effect of starvation and CDT for the tumor treatment.

Lactic acid is considered a metabolic waste product of glycolysis in tumor cells. However, lactic acid accumulation-induced tumor angiogenesis, metastasis, and immunosuppression could promote tumor growth and invasion. Therefore, lactic acid is considered to be an effective target for regulating the abnormal metabolism of TME by blocking production or direct depletion. In addition, ATP is the most direct energy source in living organisms. By inhibiting the production of ATP, the energy demand of cancer cells can be blocked, which is an effective way to improve the effectiveness of cancer treatment 180. In a recent study, nanoagents that self-supply O_2_ are designed to enhance CDT/starvation therapy by producing •OH, depleting lactic acid and ATP 181. In this therapeutic system, the core of perfluorooctyl bromide (PFOB) nanodroplets was coated with LOX-loaded TA-Fe (III) complex layer (PFOB@TA-Fe (III)-LOX, PTFL) (**Figure 8B**). After being internalized by tumor cells, O_2_-self-sufficient PTFL NPs delivered O_2_ to alleviate hypoxia, the loaded LOX catalyzed the oxidation of lactic acid with O_2_, resulting in lactic acid depletion and H_2_O_2_ production. Meanwhile, the TA-Fe (III) complex excreted intracellular ATP as an energy source and decomposed into TA and Fe (III) ions. The released Fe (III) ions were further reduced to Fe (II) ions by TA, which then catalyzed the decomposition of H_2_O_2_ into cytotoxic •OH for enhanced CDT. Another study reported a pH-responsive nanoselenium-coated manganese carbonate deposited iron oxide nanoparticles (MCDION Se) platform for combing CDT with starvation therapy 182. The system enabled enhanced synergistic effect due to H_2_O_2_ producing and ATP inhibition, and MRI could monitor the tumor treatment process.

In short, combining CDT with starvation Therapy cuts off the energy source for cancer cells and produces a significant CDT-enhancing effect by lowering pH and producing H_2_O_2_ during starvation treatment. And acidic conditions can further decompose nano-delivery systems and facilitate cascade reactions. However, the non-specific accumulation of free GOx or LOX in normal tissues inevitably limits the efficiency of CDT and enhances its cytotoxicity. Therefore, the choice of nanocarriers is very important to enhance the efficiency of this combination therapy.

### CDT in combination with GS

Gas therapy is a kind of treatment method based on special gas signal molecules, which can synergize or directly induce cancer cell apoptosis 183. Gas therapy was initially achieved by direct inhalation or drinking of gas-rich water, and later nanomedicines were developed to achieve more efficient treatments. To date, three main gas molecules, including nitric oxide (NO), carbon monoxide (CO), and hydrogen sulfide (H_2_S), have been investigated 184-186. Previous studies have shown that H_2_S and NO, as signaling molecules, can lead to vasodilation and decreased vascular tone in solid tumors, which can help alleviate tumor hypoxia and induce upregulation of H_2_O_2_ levels. Therefore, the combination of gas therapy with CDT for tumor clearance has been gradually developed in recent years.

**Figure 9:**
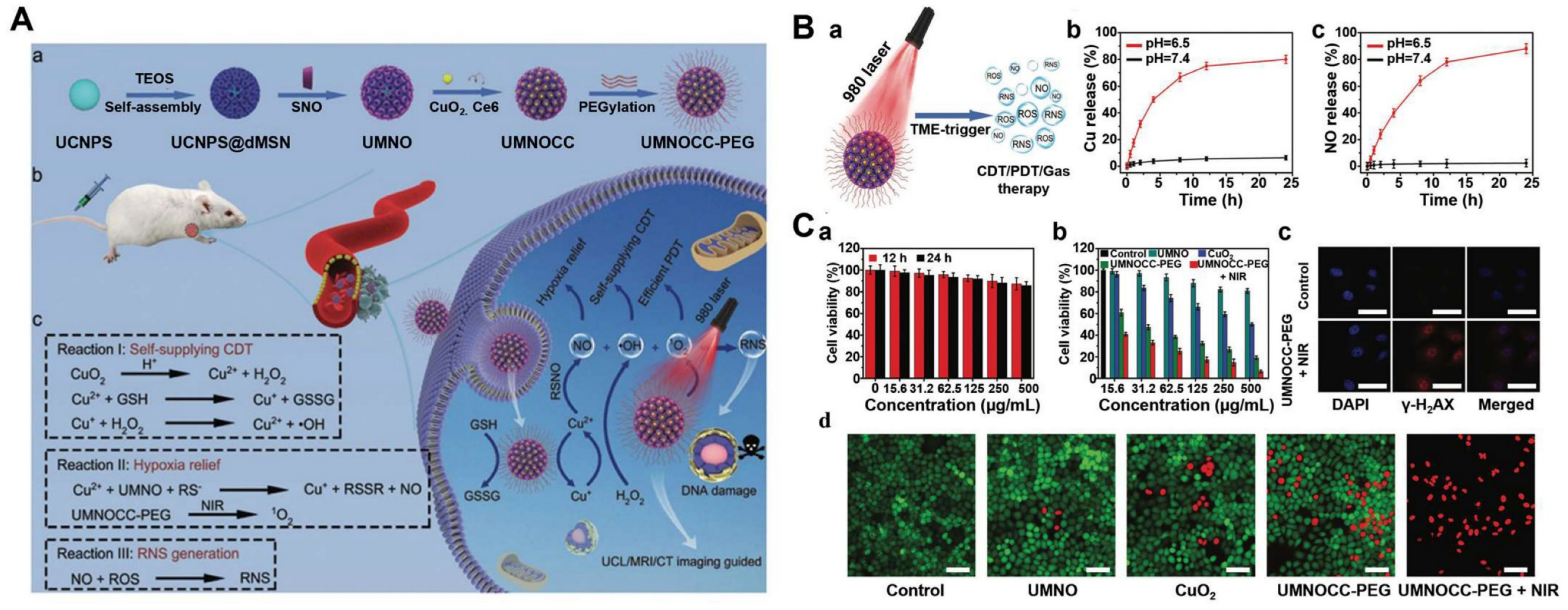
(A) a) Schematic illustration for the synthesis of UMNOCC-PEG. b) Scheme of the tumor theranostic application and (c) therapeutic mechanism of UMNOCC-PEG for the improvement of PDT induced by NIR, Cu^2+^-initiated CDT and gas therapy. (B) a) Schematic illustration of NO release from UMNOCC-PEG. b) Cumulative Cu release and c) quantitative assessment of NO generation from UMNOCC-PEG under different pH conditions. (C) a) The cell viabilities of L929 fibroblast cells upon incubation with UMNOCC-PEG for 12 h and 24 h. b) The cell viabilities of HeLa cells after being treated under different conditions. c) γ-H2AX-stained HeLa cells treated with PBS and UMNOCC-PEG (0.5 W/cm^2^, 500 μg/mL). Scale bar: 50 μm. d) CLSM images of HeLa cells co-stained with calcein AM (live cells, green) and propidium iodide (dead cells, red) after being treated under different conditions (0.5 W/cm^2^, 500 μg/mL). Scale bar: 50 μm. Reproduced with permission 187. Copyright 2020, The Royal Society of Chemistry.

NO can act as an apoptosis inducer by interacting with O_2_, superoxide ions, and transition metals to produce reactive nitrogen oxide species with high oxidative toxicity. The combination of CDT and NO-assisted gas therapy could increase the killing effect of cancer cells. For example, Yang et al. coated a mesoporous silica layer on the surface of upconversion NPs to increase the loading capacity of NO donors and subsequently packed copper peroxide nanodots, Ce6 and PEG into the silica pores to obtain UCNPs@dMSN-SNO@CuO_2_-Ce6 PEG (UMNOCC-PEG, **Figure 9A**) 187. After endocytosis by cancer cells, copper peroxide nanodots decomposed in acidic TME and generated large amounts of copper ions and H_2_O_2_ and substantial NO for enhanced CDT treatment and gas therapy, respectively. Importantly, the simultaneous release of ROS and NO ensured efficient production of RNS, which could directly induce DNA damage by triggering free radical peroxidation. In addition, UMNOCC-PEG was able to alleviate hypoxia and deplete GSH, thereby enhancing the efficacy of PDT. As shown in **Figure 9B (a-c)**, the release of copper ions was as high as 80% at pH 6.5. Notably, the presence of copper ions allows UMNOCC-PEG to have a relatively robust and rapid release of NO at pH 6.5, leading to enhanced NO therapy. In vitro, UMNOCC-PEG + NIR showed the highest therapeutic efficacy, significantly reducing HeLa cell survival rate to 6.4%. In addition, cells treated with UMNOCC-PEG+NIR lasers showed a distinct γ-H2AX fluorescence signal within the nucleus compared to the control group, suggesting that UMNOCC-PEG+NIR enhanced DNA damage (**Figure 9C, a-c**). Then, cell dead-live staining showed that almost all cells died in the UMNOCC-PEG+NIR group, indicating that UMNOCC-PEG enabled synergistic CDT/PDT/NO treatment (**Figure 9C, d**). Additionally, SNP anion ([Fe-CN)_5_NO^]2-^), as a GSH-responsive NO donor, can release NO upon activation of GSH in tumor cells. Zhou et al. successfully synthesized SNP/MgMnFe-LDH (S2MFL) supramolecular nano-reagent containing both Mn^2+^ and SNP anions, and the synergistic effect of Mn^2+^ and SNP can produce high levels of ONOO^-^ to cause DNA fragmentation for synergistic CDT/NO anti-tumor 188.

Previous studies have shown that H_2_S induced an upregulation of H_2_O_2_ levels, which was highly beneficial in enhancing the therapeutic effect of CDT. Combining CDT and H_2_S therapy into a single nanoplatform will achieve more effective anti-cancer effects. For instance, Cai et al. successfully prepared FeS@BSA nanoclusters based on the self-assembly of ferrous sulphate and BSA to form H_2_S-enhanced CDT system 186. Under an acidic environment, FeS@BSA degraded to release H_2_S and Fe^2+^. The released H_2_S gas inhibited catalase activity in cancer cells to promote H_2_O_2_ levels, which further enhanced the Fe^2+^-mediated Fenton reaction, and significantly improved intracellular ROS production. The results confirmed that the treatment system could achieve superior anti-tumor performance under MRI guidance. In another study, Huang et al. used BSA as a template to synthesize size-controlled and biodegradable sub-stable γ-phase manganese sulfide nanomaterials (named MnS@BSA) for gas therapy-induced CDT 189. At pH 6.8, H_2_S was released for gas therapy, and manganese ions were released for CDT and MRI. *in vivo* experiments have shown that intravenous injection of MnS@BSA significantly inhibited tumor growth and greatly prolonged the survival of tumor-bearing mice.

Additionally, hydrogen therapy has also shown excellent therapeutic properties for many diseases, including cardiovascular disease, neurodegenerative disease, and cancer 190. To avoid the limitations caused by poor targeting, high diffusivity, and low solubility of hydrogen, a H_2_ self-generated nanoplatform through in situ water splitting induced by NIR laser was developed. The core-shell structure nanoplatform (CSNPs) was composed of NaGdF_4_: Yb, Tm/g-C_3_N_4_/Cu_3_P (UCC) and ZIF-8, and further modified with the targeted molecule folic acid 191. CSNPs were selectively captured by tumor cells due to acid-responsive ZIF-8 shell, EPR effect and folate receptor-mediated endocytosis. Z-scheme heterojunction g-C_3_N_4_/Cu_3_P generated H_2_ and ROS for H_2_ therapy and PDT under 980nm laser irradiation. In addition, Cu (I) in Cu_3_P could convert H_2_O_2_ into •OH for CDT through a Fenton reaction. Besides, the local temperature rising in the tumor area increased the Fenton reaction rate of Cu(I) with H_2_O_2_. Additionally, GSH in tumor TME could reduce Cu (II) to Cu (I), ensuring a continuous Fenton reaction. Moreover, photogenerated electrons in g-C_3_N_4_ also oxidized GSH to GSSG to reduce the antioxidant capacity of tumors. Thus, CDT will have a better therapeutic effect. Particularly, hydrogen therapy has an excellent potential for reducing tissue inflammation caused by oxidative stress in PDT or excessive heat in PTT 192,193. Therefore, CDT combined with phototherapy and H_2_-assisted gas therapy can produce better tumor treatment results.

In conclusion, the combination of gas therapy and CDT can significantly improve oxidative stress or nitrosative stress to effectively kill the tumor. CDT/gas therapy also reduce the amount of toxic gas to reduce its side effects on normal tissues. As a "green" therapy, gas therapy has been recognized as a new cancer treatment strategy. However, the limitations of gas therapy, such as the short half-life of gases, the instability of gas donors, and the controlled release of gases, also put forward high requirements for the design of carrier for synergistic therapy. Moreover, the biological effect of gas therapy is largely dependent on the treatment site, concentration, and duration. Thus, it is important to monitor their concentration and biodistribution in the body. The development of multifunctional nanosystems that integrate diagnostic and therapeutic functions to optimize cancer treatment deserves full recognition.

### CDT in Combination with GT

**Figure 10:**
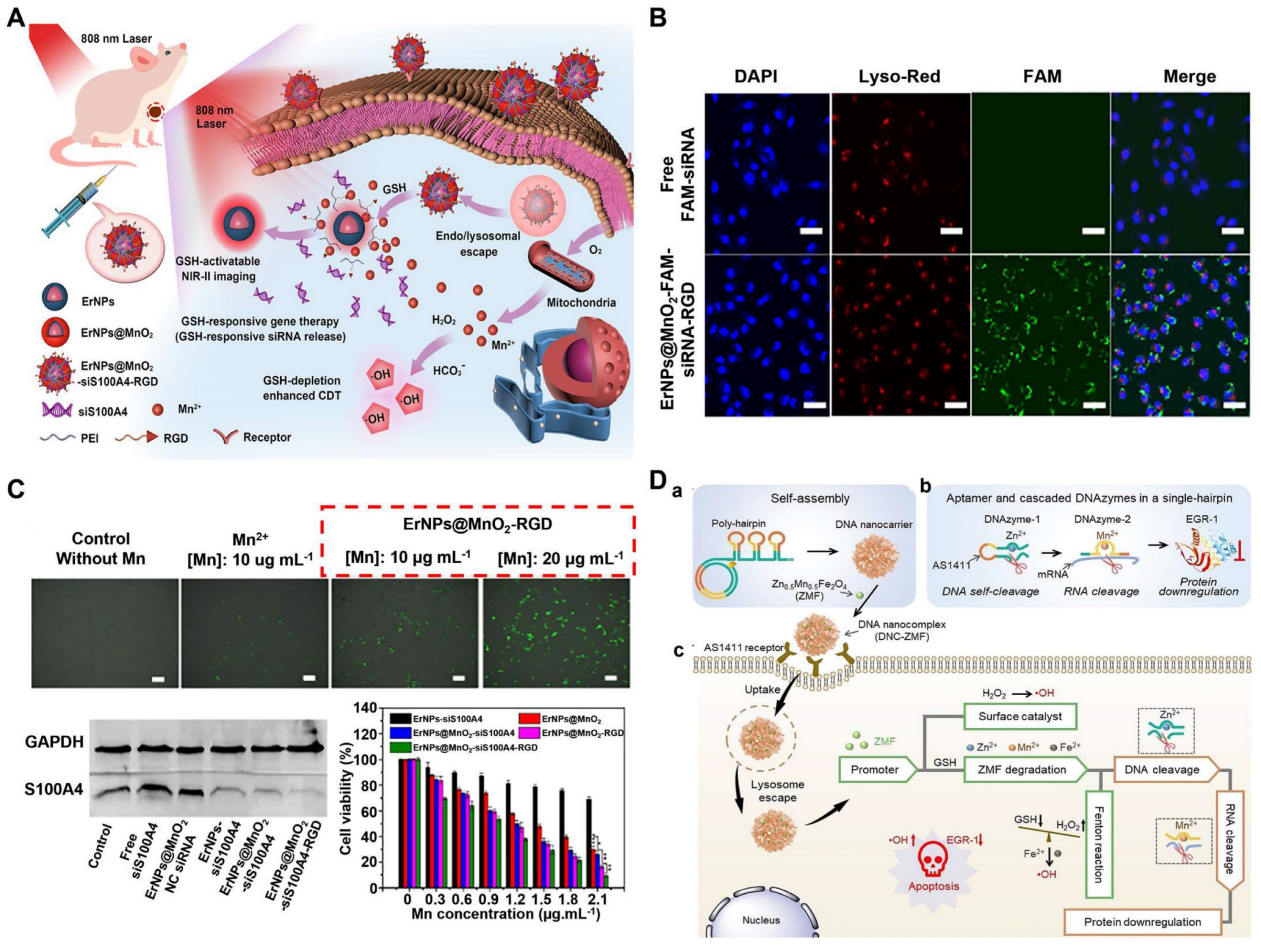
** (**A) Schematic illustration of GSH stimuli-responsive lanthanide-doped NIR-II luminescent nanoprobes for TNBC precise gene therapy in synergism with CDT. (B) CLSM images of MDA-MB-231 cells incubated with free FAM-siRNA or ErNPs@MnO_2_-FAM- siRNA-RGD for 4 h (scale bar: 100 μm). (C) a). Intracellular detection of •OH produced by Mn^2+^ and ErNPs@MnO_2_, and green fluorescence represents the generated •OH (scale bar: 100μm). MDA-MB-231 cells treated with different concentrations of MnCl_2_ (10 μg/mL) of Mn and ErNPs@MnO_2_ (10, 20 μg/mL of Mn), respectively, b). Expression of S100A4 in the MDA-MB-231 cells determined by western blot analysis, c). Cytotoxicity of ErNPs-siS100A4, ErNPs@MnO_2_, ErNPs@MnO_2_-siS100A4, ErNPs@ MnO_2_-RGD, and ErNPs@MnO_2_-siS100A4-RGD in MDA-MB-231 cells (n = 4, *p < 0.05, **p < 0.01, and ***p < 0.001). Reproduced with permission 197. Copyright 2021, American Chemical Society. (D) Molecular design of DNA nanocomplex containing cascade DNAzymes and promoter-like Zn-Mn-Ferrite (ZMF) for combined gene/chemo-dynamic therapy. (a) Construction of DNA nanocomplex (DNC- ZMF). (b) Cascade catalytic cleavage of DNAzymes. (c) Proposed antitumor mechanism (intracellular catalytic activity and chemical reactions) of DNC-ZMF in the combined gene/chemo-dynamic therapy. Reproduced with permission 204. Copyright 2022, Wiley-VCH.

Gene therapy is a method of transforming exogenous therapeutic genes into cells, and then changing the original gene expression of cells to treat diseases through the transcription and translation of exogenous genes 194. Nearly fifteen years ago, gene therapy was first proposed for the treatment of inherited single-gene diseases. Soon after, its potential applications extended to diseases such as cancer 195. Gene therapy is an early-stage cancer treatment with high efficacy and few side effects. The shortcomings of current gene-based therapies emphasize the need for a multifunctional vector, which could promote efficient cellular uptake of nucleic acid reagents and enable combination therapy of multiple therapeutic modalities. Small interfering RNA (siRNA)-mediated gene therapy works by specifically silencing the expression of oncogenic target genes, possessing low cytotoxicity and superior therapeutic outcome 196. Recently, Zhang et al. prepared a MnO_2_-coated rare earth nanoprobe (ErNPs@MnO_2_-siS100A4-RGD) 197 as siS100A4 carrier, and then modified the nanoprobe with the polypeptide Arg-Gly-Asp (RGD) to ensure precise tumor targeting property (**Figure 10A**). MnO_2_ acted as a vector for siRNA delivery and reacted with GSH in the TME, which greatly enabled precise gene therapy for tumors and achieved GSH-depletion-enhanced CDT by Mn^2+^ mediated Fenton-like reaction. Lanthanum fluorescent nanoprobes have been extensively developed and studied to precisely target tumors' localization 198. In this system, researchers combined ErNPs with MnO_2_ to further construct a TME-activated tumor localization nanoplatform. S100A4 is one of the proteins closely related to breast cancer growth and metastasis 199. siS100A4 degraded the mRNA of S100A4 and inhibited the expression of the corresponding protein, which limited tumor growth and metastasis. After internalization, siS100A4 nanodrugs could be dissolved under GSH, releasing Mn^2+^ ions and siRNA. **Figure 10B** showed ErNPs@MnO2-FAM-siRNA-RGD diffused into the cytoplasm, while cells incubated with free FAM-siRNA showed almost no green fluorescence. **Figure 10C, a** showed that MDA-MB-231 cells treated with ErNPs@MnO_2_-siS100A4-RGD produced higher •OH than in the control group, and the results of MDA-MB-231 western blot in **Figure 10C, b** further confirmed the downregulation of S100A4 in MDA-MB-231 cells. MTT analysis of MDA-MB-231 cells after exposure to PBS, siS100A4 alone, and ErNPs@MnO_2_-siS100A4-RGD showed the synergistic effect between CDT and gene silencing was more effective in killing tumor cells (**Figure 10C, c**). Another study designed a siRNA-modified amorphous iron oxide (AIO) nanodrug with modulated TME. siRNA was encapsulated within nanoparticles to improve its system stability, while the nanoparticles surface was modified with PEG encapsulation to prolong blood circulation and was not easily recognized by mononuclear phagocyte systems in the liver and spleen. siRNA achieved endosomal escape by osmolality and/or endosomal membrane oxidation induced by iron ions released by AIO. This gene therapy led to acidosis-induced tumor cell death by modulating the glycolytic pathway based on silencing carboxylic acid transporters (MCTs), and the released iron ions would react with H_2_O_2_ to produce •OH through a Fenton-like reaction. Notably, the silencing of MCT4 blocked the outflow of intracellular lactic acid, which further stimulated more production of H_2_O_2_ to amplify the Fenton-like reaction and oxidative damage to tumor cells, so as to achieve effective CDT/gene combination therapy 200.

In recent years, DNA nanomaterials have been used as therapeutic vectors for precise cancer treatment 201. DNAzyme is a single-stranded DNA having various catalytic functions that can precisely catalyze the cleavage of DNA or RNA in cells, down-regulate disease-related proteins, and thus exert therapeutic effects 202. However, the efficiency of DNAzyme depends heavily on delivery systems and metal ions that act as cofactors to activate catalytic activity 203. In a recent study, Yang et al. reported a DNA nanocomplex (DNC-ZMF) that contained cascading DNAzyme and promoter-like zinc-manganese ferrite (ZMF) for combining gene therapy/CDT. In addition, AS1411 aptamers were integrated into DNA sequences to achieve specific tumor targeting, increased cell uptake and reduced systemic toxicity (**Figure 10D**). The nanocomposite targeted and entered tumor cells by recognizing nucleolin. It was then decomposed by H^+^ and GSH in the tumor and further released Zn^2+^ and Mn^2+^ to initiate subsequent combination therapy: DNAzyme-1 catalyzed the self-breaking of ultra-long DNA strands with Zn^2+^ as a cofactor, producing fragments containing DNAzyme-2; DNAzyme-2 catalyzed the lysis of mRNA with Mn^2+^ as a cofactor, resulting in the down-regulation of early growth reaction protein 1 (EGR-1), thereby inhibiting the proliferation of tumor cells and promoting their apoptosis. Moreover, Zn^2+^, Mn^2+^, and Fe^2+^ induced the catalytic generation of •OH by the Fenton reaction, which was the CDT process. In particular, the sequential cascade enzyme reaction initiated by the two DNAzymes maximized the therapeutic function of DNAzyme, thereby realizing low-toxicity and efficient combination therapies to eradicate tumors 204. Furthermore, Chen et al. formed the hybrid nanoparticles (Cu-Dzy) through the interaction of DNAzyme and Cu^2+^ coordination, which had ultra-high loading capacity and could effectively co-deliver DNAzyme and Cu^2+^ into cancer cells for combined therapy. In order to improve the stability of Cu-Dzy NPs in the physiological environment, they were coated with a thin layer of the metal phenolic network to generate the final nanostructure (Cu-Dzy@TA). After entering cancer cells, the DNAzyme released from hybrid NPs silenced human vascular endothelial growth factor-2 (VEGF2) mRNA for gene therapy. Meanwhile, GSH reduced Cu^2+^ to Cu^+^, which catalyzed endogenous H_2_O_2_ to generate high toxicity •OH for CDT. In summary, the specific gene silencing, GSH depletion, and •OH generation induced amplified cascade antitumor effects 205.

In conclusion, designing gene therapy platforms with the ability to reduce the pH of TME is a viable strategy to enhance the effect of CDT. The RNAi NP platform can reconstruct acidic TME by MCT-4 silencing, optimize the catalytic efficiency of Fenton/Fenton-like reactions, and ultimately improve the anti-cancer efficiency of CDT. However, the efficient and sustained expression of exogenous genes in cells is the key to the success of gene therapy, which is closely related to the selection of vectors.

### CDT in combination with OT

Oncosis is defined as cytoplasmic swelling and nuclear lysis, in which the integrity of the cell membrane is destroyed, and DNA is broken into nonspecific fragments, resulting in cell lysis and inflammatory reaction 206, 207. Studies have shown that the interaction of dihydroartemisinin (DHA) with ferrous ions could generate ROS, which will avoid the restrictions of exogenous conditions, endogenous H_2_O_2_, and strict Fenton's reaction conditions 208. However, lower intracellular Fe^2+^ concentrations may be an obstacle to this strategy. To overcome this limitation, Prof. He developed a multifunctional nanomaterial that could co-deliver DHA and Fe to the tumor site, then induced effective CDT combined with chemotherapy and OT 209. The mesoporous silica nanoparticles were prepared as carriers, doped with calcium and iron to obtain CFMSN, and loaded with DHA for constructing CFMSN@DHA. After entering cancer cells, CFMSN@DHA with catalytic activity could directly convert intracellular H_2_O_2_ into highly toxic •OH for CDT. Meantime, the acidic tumor microenvironment triggered the release of Ca^2+^ and Fe^3+^, which caused the particles to collapse, and was accompanied by DHA release for chemotherapy. The released Fe^3+^ was further reduced to Fe^2+^ by high concentrations of intracellular GSH. After that, the produced Fe^2+^, on the one hand, activated the Fenton reaction to produce •OH. On the other hand, cleaved the peroxide bond of DHA to generate free radicals. Finally, the increasing levels of ROS in cells significantly amplified the efficacy of CDT. In addition, the release of Ca^2+^ caused overload for OT. Interestingly, DHA inhibited intracellular Ca^2+^ efflux and maintained high cytosolic Ca^2+^ levels to enhance Ca^2+^-mediated OT. Thus, CFMSN@DHA has produced high anti-cancer effects through chemotherapy, CDT, and OT.

**Figure 11:**
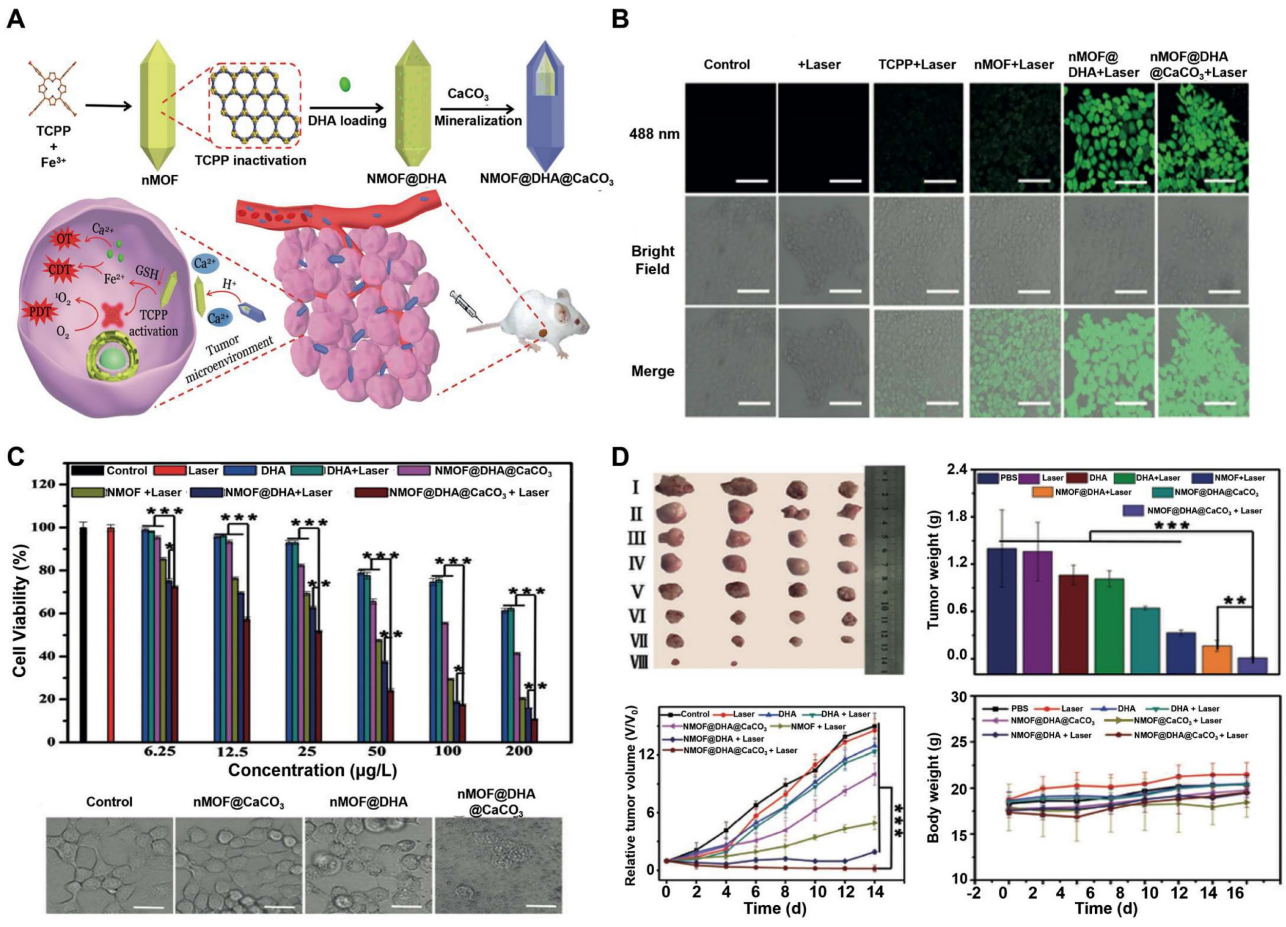
(A) Schematic illustration of the preparation of the nanoplatform and the programmed drug release for cancer therapy. (B) Confocal fluorescence imaging of 4T1 cells treated under different conditions to evaluate ROS production based on DCFH-DA fluorescence intensity. The nanoparticles were incubated with 4T1 cells for 4 h, then DCFH-DA probes were incubated for 20 min with 4T1 cells before irradiation. Scale bars are 100 mm. (C) *in vitro* therapy effect. (a) MTT assay of 4T1 cells with different treatments at different concentrations. (b) Morphology of 4T1 cells with different treatments. Scale bars are 25 mm. (*P < 0.05, **P<0.01, ***P<0.001). (D) *in vivo* anticancer effect. a) Picture of tumors dissected on the 14th day after different treatments (I: PBS, II: Laser, III: DHA, IV: DHA + Laser, V: NMOF@DHA@CaCO_3_, VI: NMOF + Laser, VII: NMOF@DHA + Laser, and VIII: NMOF@DHA@CaCO_3_ + Laser). b) Average tumor weights in different treatment groups. c) Tumor growth curve after the mice received different treatments. d) Body-weight changes within 14 days during treatment. (*P < 0.05, **P < 0.01, ***P < 0.001). Reproduced with permission 210. Copyright 2019, Wiley-VCH.

To avoid DHA leakage from the carrier, which may lead to limited efficacy and severe side effects, Tang and his colleagues developed a drug delivery and programmed release platform (NMOF) based on Fe_3_O(OOC)_6_ metal clusters and 4,4,4-(porphyrin-5,10,15,20-tetradecyl) tetrabenzoic acid (TCPP). Then DHA was encapsulated within the pores of the NMOF, and then the CaCO_3_ mineralized layer was further coated on the NMOF by biomineraliation to form NMOF@DHA @CaCO_3_ 210. This drug delivery platform could prevent drug leakage during *in vivo* transportation and enable site-specific release of DHA at the tumor site. When the prepared nanomedicine reached the tumor site, the outer CaCO_3_ mineralized layer dissolved in the weakly acidic microenvironment of the tumor site, generating NMOF@DHA and Ca^2+^ ions. The Fe^3+^ ions in NMOF were then reduced to Fe^2+^ by the high concentration of GSH in cancer cells, resulting in complete structural collapse, leading to the release of DHA and activation of the photosensitizer TCPP, thereby enabling synergistic Fe^2+^-DHA-mediated CDT, Ca^2+^-DHA-mediated OT, and TCPP-mediated PDT (**Figure 11A**). As shown in **Figure 11B**, the NMOF@DHA @CaCO_3_ +Laser group produced a large amount of ROS in 4T1 cells, as demonstrated by fluorescent probe DCFH-DA staining. The lowest cell viability in the NMOF@DHA@CaCO_3_ +Laser group was confirmed by MTT assay. Bright field images of 4T1 cells from different treatments showed that the cells underwent oncosis due to the introduction of DHA and Ca^2+^ (**Figure 11C, a-b**). The significant anti-cancer effect of the NMOF@DHA@CaCO_3_ +laser group *in vivo* was confirmed by monitoring tumor growth in 4T1 tumor-bearing mice (**Figure 11D, a-d**). The above results indicated that the prepared nanoplatform was a safe and effective drug delivery system.

### CDT-based other combination cancer therapy

**Figure 12:**
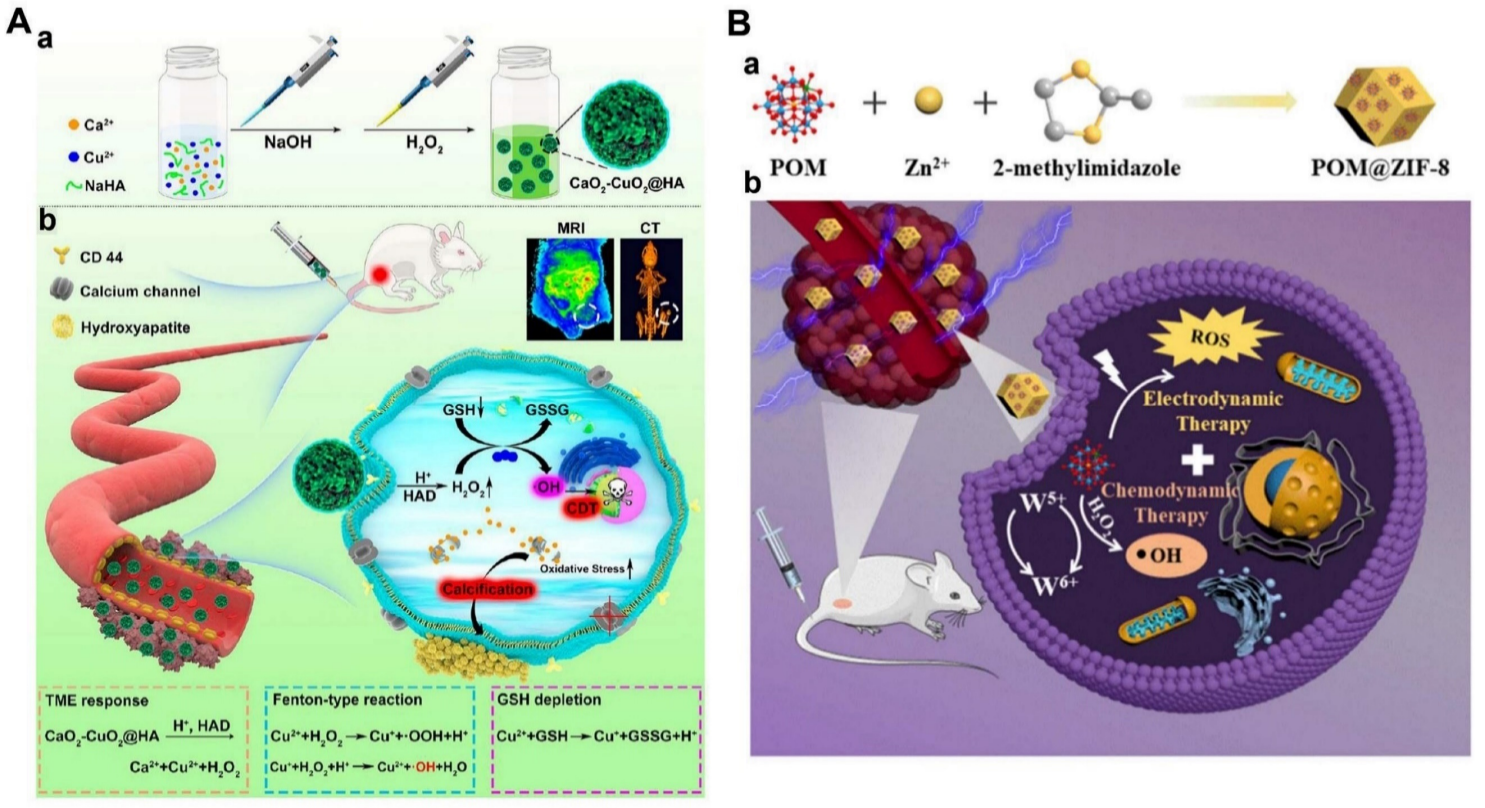
(A) Schematic illustration of the synthesis and antitumor performance of CaO_2_-CuO_2_@HA NC. Reproduced with permission 212. Copyright 2022, American Chemical Society. (B) Schematic illustration of POM@ZIF-8 NPs for the synergistic electrodynamic/chemodynamic tumor treatment; a) Preparation of POM@ZIF-8 NPs; b) Anticancer mechanism of POM@ZIF-8 NPs. Reproduced with permission 214. Copyright 2022, American Chemical Society.

CDT combined therapy has made significant progress in the field of anti-tumor, and each treatment has its advantages and disadvantages (**Figure 15**). In addition to the CDT-based combination strategy described above, other combined strategies have been developed. Recently, Professor Bu's group described that bioactive ions are involved in many important processes, including osmotic pressure and acid-base balance maintenance, catalysis and signaling pathway activation, protein and enzyme composition, and biomolecular targeting. Their abnormal distribution/accumulation in cells may lead to irreversible damage or biochemical reactions that activate the production of cytotoxic components, leading to cell death. This action of ions could be used as a new oncology treatment technique called ion interference therapy 211. In order to improve the anti-tumor efficiency of CDT, a lot of efforts have been made to increase the level of H_2_O_2_. As an efficient H_2_O_2_ self-supply, various metal peroxide nanomaterials have attracted more and more attention in enhancing tumor treatment. However, a single metal peroxide is insufficient to achieve more effective anti-tumor properties. Here, Yang et al. synthesized hyaluronic acid-modified calcium peroxide and copper peroxide nanocomposites through a simple one-step strategy (**Figure 12A**). Due to the EPR effect and the specific recognition of hyaluronic acid with the CD44 protein on the surface of tumor cells, nanodrugs effectively accumulated at the tumor site, then large amounts of Ca^2+^, Cu^2+^, and H_2_O_2_ could be released simultaneously in TME. With the help of glutathione depletion, a large number of •OH were produced by the enhanced Fenton reaction between Cu^2+^ and self-sufficient H_2_O_2_. Moreover, overloaded Ca^2+^ could lead to mitochondrial damage, which enhanced oxidative stress in tumor cells. In addition, the imbalance of calcium transport channels caused by oxidative stress could further promote tumor calcification and necrosis, so the synergistic effect of CDT caused by Cu^2+^ and IIT caused by Ca^2+^ show more satisfactory antitumor efficiency than single treatment 212.

Electrodynamic therapy, as a new dynamic treatment strategy, can generate •OH under electric stimulation, showing obvious advantages in bacterial infection and clinical transformation 213. Recently, Professor Wang's team reported the results of electrodynamic therapy combined with CDT in the treatment of cancer. Polyoxometalate-modified zeolitic imidazolate framework-8 nanoparticles (POM@ZIF-818-21 NPs) were designed and synthesized (**Figure 12B**). In acidic TME, POM acted as an electrosensitizer to generate ROS in an electric field environment. At the same time, POM can further release W^5+^ by catalyzing the decomposition of H_2_O_2_ under acidic conditions and producing •OH through Fenton-like reactions. In this work, POM@ZIF-8 NP was shown to have significant antitumor efficacy* in vitro* and* in vivo* 214.

Enhanced EDT is achieved by the rational design of nanoelectrosensitizers. In most cases, Pt nanoparticles are used as nanoelectrosensitizers for EDT-mediated tumor therapy 215. However, the potential functional mechanism of nano-electrosensitizers to enhance catalytic activity remains to be further studied. In addition, the application of EDT is limited to tumor treatment and antibacterial wound dressings, and there is a great need to explore more details based on multiple disease models.

### CDT in combination with trimodal therapy

Compared with two-modal combination therapy, three-modal synergistic therapy can achieve higher antitumor effects with reduced dosages administered. So far, a large number of studies have been reported on three-modality combination therapy, such as the synergistic effect by CDT-phototherapy-immunotherapy trimodal therapy, which has been partially demonstrated above 159, 163. Other CDT-based combination therapies include CDT-chemotherapy-phototherapy 216, CDT-SDT-chemotherapy 217, CDT-chemotherapy-MHT 218, CDT-starvation therapy-PTT 219, CDT- PTT-RT 220. In this section, we will highlight several representative examples to illustrate the enhanced therapeutic efficacy of CDT-based trimodal cancer therapy.

**Figure 13:**
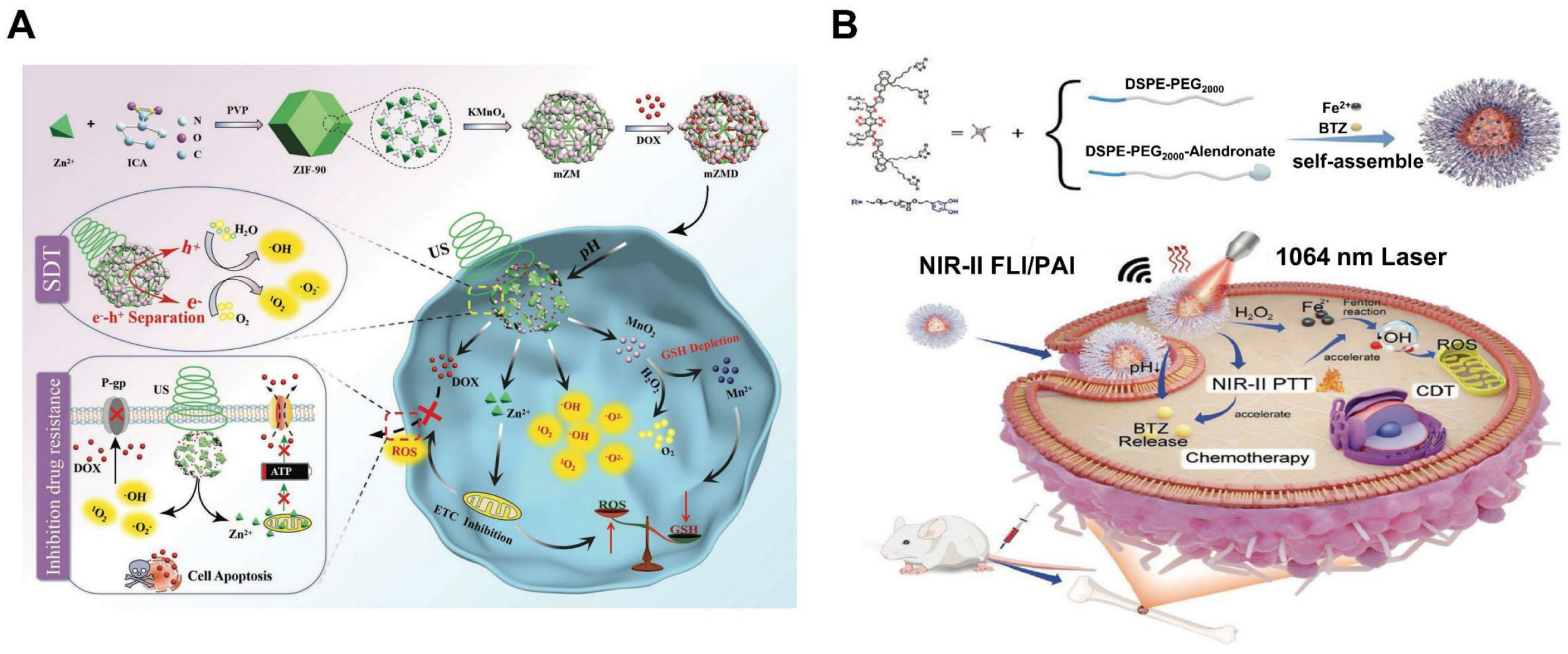
(A) Synthetic process and mechanism diagram of enhancing SDT and overcoming cancer drug resistance of mZMD. Reproduced with permission 217. Copyright 2022, Wiley-VCH. (B) Schematic illustration of 1064 nm light-activated NIR-II phototheranostic nanoplatform (BTZ/Fe2+@BTF/ALD) for combined NIR-II PTT/chemotherapy/CDT of breast cancer bone metastases. Reproduced under the terms of the http://creativecommons.org/licenses/by/4.0/ License 221, published by WILEY-VCH Verlag GmbH & Co. KGaA, Weinheim.

One example is CDT-SDT-chemotherapy based trimodal therapy 217. As emerging non-invasive treatment modalities, SDT and CDT are often limited by the hypoxic environment of tumors. Here, Liu et al. constructed degradable therapeutics (ZIF-90@MnO_2_/DOX, mZMD NCs) based on mesoporous zeoliteimidazole salt backbone @MnO_2_/doxorubicin hydrochloride nanocomposites, which achieved enhanced SDT/CDT/chemotherapy by modulating TME and overcoming drug resistance (**Figure 13A**). When mZMD accumulated to the tumor site, they broke down and released DOX in response to US irradiation and TME. MnO_2_ not only oxidized GSH to enhance oxidative stress, but also converted endogenous H_2_O_2_ to O_2_ to improve hypoxic TME, enhancing chemotherapy for Dox and providing substrates for the SDT. At the same time, the generated Mn^2+^ promoted the separation of e^-^-h^+^ under US radiation for enhanced SDT and catalyzed endogenous H_2_O_2_ to •OH for CDT. The mZM composite structure reduced the US-induced recombination rate of e^-^ and h^+^ to further improve SDT. In addition, the released Zn^2+^ inhibited electron transport chain, which disrupted the redox balance of TME and inhibited the production of ATP and the expression of P-glycoprotein (P-gp), further overcoming the resistance of cancer cells to DOX. The results show that mZMD NCs achieve the synergistic effect of enhancing SDT/CDT/chemotherapy and greatly inhibited the growth of tumor cells. In addition, mZMD NCs had good biocompatibility and biosafety. Therefore, smart biodegradable mZMD provides new insights for developing biomaterials for ROS-related therapeutic strategies.

In order to improve the therapeutic effect of bone metastases, the current treatment strategy mainly focuses on the combination of chemotherapy and phototherapy, in order to overcome the limited tissue penetration depth of NIR-I light, Professor Fan et al. jointly reported a phototherapy nanoparticle (BTZ/Fe^2+^@BTF/ALD) for NIR-II fluorescence imaging and NIR-II photoacoustic imaging-guided NIR-II PTT, chemotherapy and CDT of breast cancer bone metastasis 221. This phototherapy was performed by integrating dopamine-modified NIR-II absorption of donor-receptor-donor small molecules (BBT-FT-DA), borate anticancer drug Bortezomib (BTZ), and Fe^2+^ as CDT catalysts into a bone-targeted ligand alendronate modified amphiphilic pegylated phospholipid. In acidic and H_2_O_2_ overexpressed TME, boric acid-catechol bonds are severed and BTZ and Fe^2+^ ions are released to initiate chemotherapy and CDT. Using the mouse 4T1 bone metastasis model, BTZ/Fe^2+^@BTF/ALD significantly inhibited the progression of tumor cells in bone tissue by synergistic NIR-II PTT/chemotherapy/CDT effect (**Figure 13B**). Overall, this work provides new insights to guide the development of NIR-II phototherapy in the treatment of bone metastases in breast cancer.

### CDT in combination with multimodal therapy

The heterogeneity, complexity, and diversity of malignancies often result in unsatisfactory treatment outcomes, while bimodal and trimodal treatment strategies can partially alleviate this dilemma. However, bimodal and trimodal therapies with low risk of recurrence, no drug resistance, and worse efficacy still remain a great challenge 222, 223. Therefore, CDT-based combination therapies by integrating four or more therapies are considered a good option in the fight against cancer.

**Figure 14:**
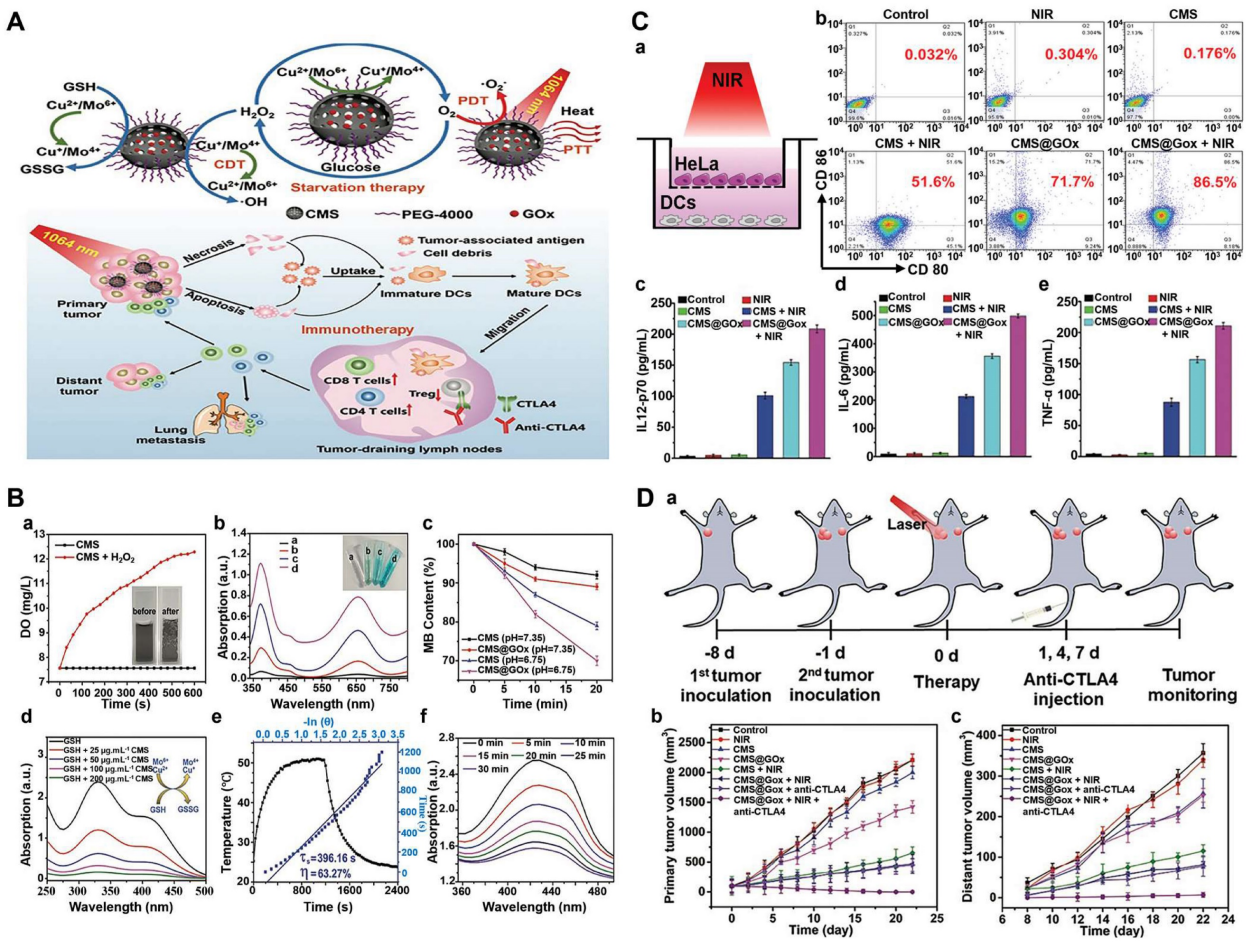
(A) Schematic illustration of fabrication and mechanism of PEGylated CMS@GOx for PTT/PDT/CDT/starvation therapy. The mechanism of antitumor immune responses induced by PEGylated CMS@GOx-based phototherapy in combination with checkpoint blockade therapy. (B) a) O_2_ generation curve of CMS aqueous solution (200 μg/mL, pH = 6.75) without and with H2O2 addition (100 × 10^-6^ m). Illustrations are O_2_ generation photographs of CMS with H_2_O_2_ addition before and after 1 h; b) H_2_O_2_ generation in CMS@GOx solution arising from the addition of different concentrations of glucose, a: 0 × 10^-3^ m, b: 2 × 10^-3^ m, c: 4 × 10^-3^ m, d: 8 × 10^-3^ m; c) Degradation of MB over CMS or CMS@GOx (200 μg/mL) due to the generation of •OH in 1 mg/mL of glucose solution containing H_2_O_2_ (100 × 10^-6^ m) at different pH values; d) GSH deple- tion (89 × 10-6 m) under the reduction of different concentrations of CMS; e) Heating and cooling curves of CMS aqueous solution (200 μg/mL, 1 mL) under 1064 nm (0.48 W/cm^2^) laser irradiation, linear time data obtained from the cooling period; f) Depletion of DPBF over CMS due to •O2- generation (65 μg/mL of CMS, 0.48 W/cm^2^ of 1064 nm laser). (C) a) A scheme illustrating the transwell insert system; b) Quantification of CD80 and CD86 expression on the surface of human bone- marrow-derived DCs after different treatments by ow cytometry; c-e) The secretion levels of IL-12p70, IL-6, and TNF-α by ELISA assay in DC suspensions after different treatments. (D) a) Scheme of PEGylated CMS@GOx-based synergistic comprehensive treatment combined with anti-CTLA4 checkpoint blockade treatment for bilateral tumor models in Balb/c mice. b, c) Growth curves of primary tumor volume and distant tumor volume in Balb/c mice with different treatments. Reproduced with permission 224. Copyright 2019, Wiley-VCH.

**Figure 15:**
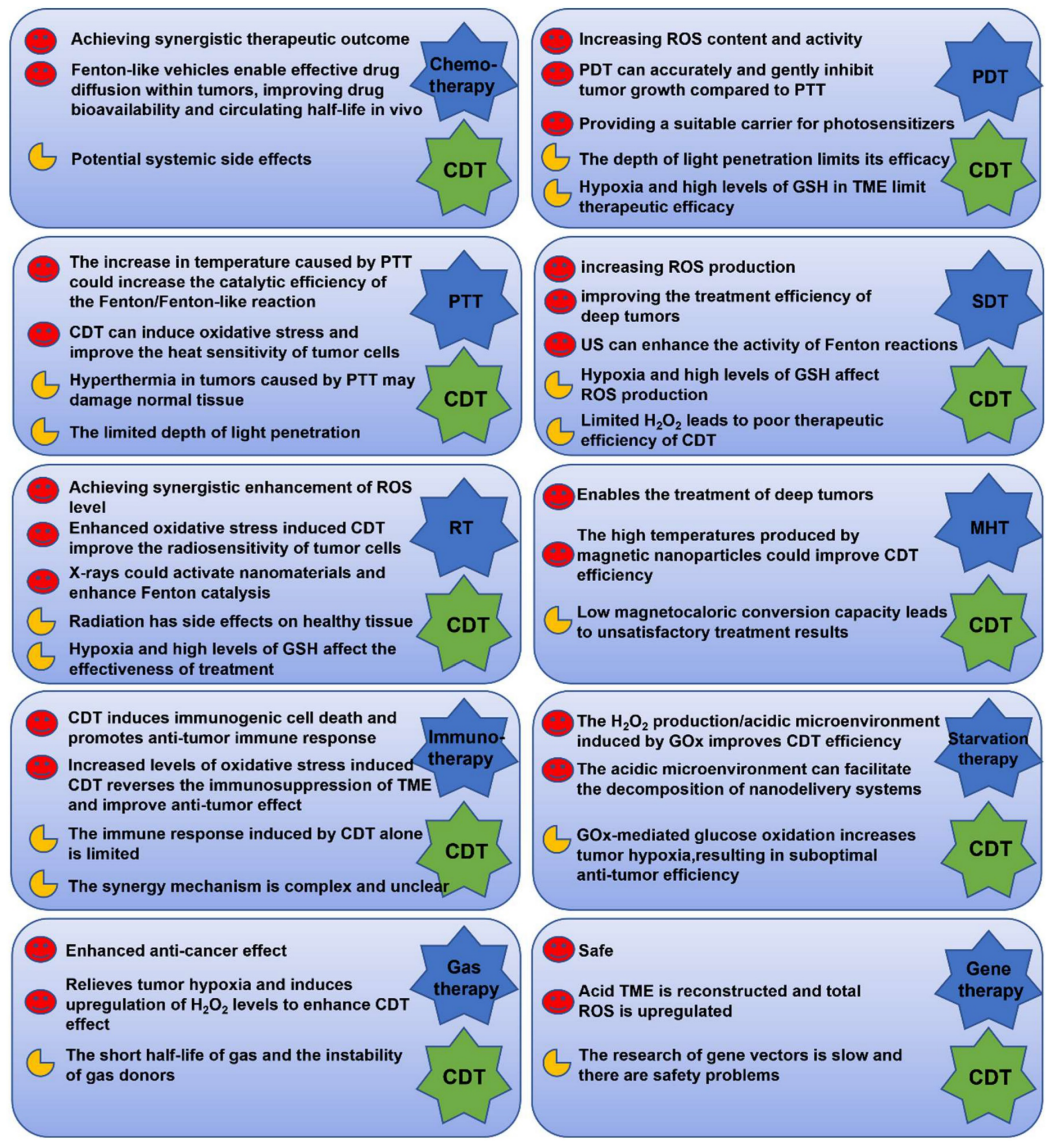
List of some types of CDT-based combination therapies and their typical features.

In a recent study, Lin et al. prepared a hollow CMS@GOx nanodrugs composed of Cu_2_MoS_4_ (CMS) and GOx, which possessed GSH peroxidase-like and catalase-like activities for depriving overexpressed GSH in the tumor environment and for generating O_2_ from endogenous H_2_O_2_ 224. The generated O_2_ activated GOx to achieve starvation therapy, and regenerated H_2_O_2_ enhanced the Cu^+^ and Mo^4+^-mediated CDT. In addition, CMS's strong absorption in the NIR showed significant PTT and PDT performance. After modification with PEG-4000, PEGylated CMS had excellent biocompatibility and overcame the immunogenicity of the host immune system. More importantly, the synergistic treatment could stimulate DCs maturation, resulting in a robust immune response, enabling CDT/PDT/PTT/starvation therapy/immunotherapy (**Figure 14A**). PEGylated CMS + H_2_O_2_ at pH 6.75 increased O_2_ content with significant bubble production. The generation of O_2_ alleviated tumor hypoxia and triggered the catalytic oxidation of glucose by GOx, resulting in the production of large amounts of H_2_O_2_ and starvation therapy mediated by intracellular glucose. At the same time, the number of regenerated H_2_O_2_ accelerated the formation of •OH efficiency by the Fenton-like reaction. In addition, CMS also exhibited good GSH consumption characteristics and high photothermal conversion performance under 1064 nm laser irradiation. It could also be used as a photosensitizer triggered by a 1064 nm laser to produce high •O2- (**Figure 14B, a-f**). Additionally, it showed that this PEGylated CMS could significantly induce DC maturation and then stimulate an immune response for efficient anti-tumor immunotherapy (**Figure 14C, a-e**). Furthermore, the *in vivo* anti-tumor results showed that PEGylated CMS@Gox + 1064 nm + anti-CTLA4 therapy could remove the primary tumor and significantly inhibit the growth of distant tumors (Figure **14D, a-c**). Therefore, combining PEGylated CMS with 1064 nm laser irradiation and checkpoint blockade therapy for synergistic treatment is beneficial for effective elimination of primary tumors and inhibition of tumor metastasis.

Multimodal therapy can integrate the advantages of multiple therapeutic approaches into a nanoplatform, the synergy between different therapies can significantly improve the treatment effect. Combined therapies can simultaneously hinder cancer cell homeostasis/metabolism at multiple target mountains, reduce dose, reduce side effects, inhibit metastatic tumors, and prevent or delay the development of acquired drug resistance. However, complex nanosystems often have side effects on normal tissues. Thus, multimodal therapy does not mean that several different treatment methods are randomly combined into a single nanoplatform. In addition, it is not advisable to assemble nanocomposites only for the purpose of multimodal therapy. The most ideal combined treatment system should give play to their unique strengths and achieve low side effects.

## Outlook and challenges

To improve the efficacy of anti-tumor drugs and reduce systemic toxicity, many therapies are combined with each other. Synergies between different therapies may improve the antitumor effects of low-dose drugs or low-power phototherapy, thereby minimizing potential toxicity to healthy tissues. Nanotechnology plays an important role in combination therapies as nanoplatforms provide carriers for integrating various formulations associated with different treatment modalities. The combination of CDT with other treatment modalities can provide the advantages of each treatment modality, resulting in additional and synergistic therapeutic effects. In addition, CDT-based multimodal therapy has broad prospects for drug resistance in the treatment of anti-multidrug resistance (MDR) and hypoxia-related tumors. Despite the enormous therapeutic potential of CDT technology, it is still in its infancy, and there are still challenges in the clinical translation process.

Currently, the biosafety of nanomaterials is a great challenge for clinical translation. CDT often releases low-cost transition metal ions with high Fenton activity to trigger the conversion of intracellular H_2_O_2_ into highly active •OH to kill tumors. However, the major problem is that exogenous administration of excessive Fenton-type heavy metals, such as iron, manganese, copper, and cobalt, may cause potential adverse effects on human health, including acute and chronic toxicity. Therefore, it is very desirable to explore new functional materials to circumvent this problem. Besides, the synthesis of nanoparticles with controllable size and shape is particularly important for their physical and chemical properties. In the traditional methods of synthesizing nanoparticles, the reaction conditions fluctuate randomly and are difficult to control. The direct consequence is that the nanoparticles have large particle size, uneven size, low encapsulation rate, single particle morphology and large batch difference. The existence of these problems has promoted the application and development of microfluidic technology in this field. Microfluidic devices can fabricate nanoparticles in a highly repeatable and high-throughput manner. Microfluidic chips can manipulate fluids in micron-scale channels, which are widely used in nanotechnology. Compared with traditional synthesis technology, the microfluidic system has the following advantages in synthesizing nanoparticles: short mixing time, small particle size, uniform size, high encapsulation rate, diverse particle morphology and good controllability. But in the application process, the challenges of manufacturing, automation and channel blockage must also be solved. Nevertheless, microfluidics still has great potential and great promise in solving the development of large-scale, cost-effective nanoparticle synthesis. Additionally, more methods are needed to comprehensively study the biosafety of nanomaterials, and a series of challenges such as the evaluation criteria of nanomaterials' therapeutic effects and the disclosure of deeper biological principles, which still need to be solved by multidisciplinary researchers.

Moreover, the development of nanocarriers with superior tumor targeting and evasion of immune system recognition in order to achieve the specificity and high efficiency accumulation of nanomedicines at tumor sites, while reducing toxicity to normal tissues remains a top priority for CDT therapy. Although there are a variety of targeted strategies that are widely used for the simultaneous delivery of multiple drugs, problems still remain. For instance, the immune rejection reaction of nanomaterials, the formation of “protein crown” during blood transport, and *in vivo* stability and targeting ability of nanomaterials. In recent years, a bionic drug delivery system combining nanoparticles and biomimetic materials has been explored for its use in tumor treatment, making nanodrugs self-identify substances and avoiding recognition by the immune system. Among the many bionic materials, the cell membrane is one of the most suitable materials that impart unique biological properties to nanoparticles. For example, the protein and glycosyl groups on the red blood cell membrane surface endow the nanoparticles with longer system retention time, less reticuloendothelial system uptake, low immunogenicity, high biocompatibility, and improved blood-brain barrier permeability, etc. Especially, cancer cell membranes rely on surface antigens with domains, which adhere to homologous cells and homologous binding proteins, providing immune evasion and homologous adhesion. These functions are difficult to fully reproduce with synthetic materials. At the same time, by modifying tumor-targeting peptides on the cell membrane surface, the enrichment of nanodrugs in the tumor can be effectively improved. However, it is particularly difficult to achieve this surface modification by commonly used chemical reaction methods, which not only damages the protein distribution of cell membranes, but also causes problems such as biological macromolecule crosslinking, protein inactivation, and poor reproducibility due to low reaction controllability, which in turn greatly reduces the practicality of this chemical targeting modification strategy. The development of genetic engineering technology provides a new way to solve the above problems, in particular, the use of CRISPR engineering technology to achieve the expression of targeted ligands on cell membranes *in vivo*. The method is simple and controllable, and the ligand's expression is stable. Through cell membrane technology, the nanomedicines can merge complex and unique surface physicochemical properties of the source cell, and the stability of the nanoparticles has significantly improved. The use of cell membrane wrapping technology facilitates the stable *in vivo* transportation of drugs and their specific release at the tumor site.

This review comprehensively summarizes the latest research in tumor chemodynamic therapy combined with other model therapies, promotes the deep integration of materials, chemistry, physics, biology, and medicine, and sets a model for cross-integration of multiple disciplines in the field of natural sciences.

## Figures and Tables

**Table 1:** Various Fenton/Fenton-like agents for CDT and strategies to enhance CDT.

Nanoplatform	Shape and size(nm)	Enhanced strategies	*In vitro*	*In vivo*	Ref, year
Aptamer/tetraoxane/ hemin	Spherical: 32.4	No dependence on either H_2_O_2_ or pH, GSH depletion	HepG2	HepG2	24, 2021
C_5_N_2_/Fe^3+^	Nanosheet,140	H_2_O_2_ production in both normoxic and hypoxic systems	4T1	4T1	25, 2022
β-cyclodextrin/ hyaluronic acid/ ferrocene-(phenyl- boronic acid pinacol ester /PA	Spherical, 250	Self-Supplying H_2_O_2_ and Self-Consuming GSH	A549	A549	26, 2022
CeO_2_/MgO_2_	Nanosheet	Boosting the H_2_O_2_ level, high pH- activated (pH = 6.5)	HeLa	HeLa	17, 2023
Vanadium-PEG	Nanosheet, 200	New catalysts, GSH consumption and O_2_ production, improving the ionization degree of the nanocatalysts through NIR laser	MCF-7	MCF-7	27, 2022
Hollow cuprous oxide@nitrogen-doped carbon	Spherical-like, 130	Lowering the GSH	4T1	4T1	28, 2022
					
Polyaniline/MoO_3-x_	nanobelts 5-10 μm to nanosheets 200-500	structural morphological transformation	4T1	4T1	29, 2023
Iron/DOX /A549cell membrane (CM).	Porous,160	single-atom catalyst, tumor targeting	A549	A549	30, 2021
PtN_4_C single-atom nanozyme	Spheroid,110	single-atom, H_2_O_2_ cycle accumulation and GSH deprivation	4T1	4T1	31, 2022
BiO_2-x_ nanosheets	Plate-shaped thickness: 6-10	Oxygen vacancy-rich	U87	U87	32, 2021
BiFe_097_Mn_0.03_O_3_	100	Electron rich	4T1	4T1	33, 2021
HULK (Liposome, laccase, MOHQ, FeCe6)	Spherical-like, 115.64 ± 1.71	accelerating the transformation of Fe^3+^ to Fe^2+^ by Light	4T1	4T1	34, 2021

**Table 2:** Combination therapies based on CDT in recent years.

Therapeutic agents	CDT agent	Material	Shape and size (nm)	In vitro	In vivo	Cell viability (c [μg/mL])	Enhanced strategies	Ref, year
**CDT- chemotherapy**								
Dox	Fe^2+^	Dox@Cu2O-PEG NCs	Spherical cluster, 78.36	MCF-7	MCF-7	15% (2)	Self-supplying H_2_O_2_	52, 2020
								
CPT	Fe^2+^/ Fe^3+^	MIL53@F-@cancer cell membranes	Hexagonal, 120	MCF-7	Hep 22	27% (100)	CPT replenishes H_2_O_2_, cancer cell membrane endows immune escaping	54, 2022
BTZ	Hemin-Zn complexes	He-Zn@HA-BTZ	Quadrilateral, 140-150	MDA-MB-231	MDA-MB-231	25% (2.5 uM)	Self-supplying Fenton catalyst and H_2_O_2_, repolarizing macrophages from M2 to M1	55, 2022
*β-*lapa	Cu^2+^	*β*-lapa@Cu-PMs	Spherical core-shell, 120	SMMC-7721, A549,4T1	SMMC-7721	10% (200)	Heightening intracellular H_2_O_2_, GSH dominated copper transfer	57, 2022
(Oxa(IV)	Mn^2+^	HSA-Oxa(IV) - HSA- Mn^2+^/GOD	Spherical, 140	4T1, MCF-7, B16F10	4T1	IC50: 4.33	The conversion of the Oxa(IV) into Oxa(II) was beneficial for the consumption of GSH, GOD upregulated the H_2_O_2_ level	58, 2022
**CDT-PDT**								
Ce6	Mn^2+^	MnO_2_-doped CeO_2_ load with Ce6	Rod, length:130 width:15	HeLa	Lewis	15.6% (Ce6: 8)	Ce6 induced oxygen starvation triggers the generation of H_2_O_2_	69, 2022
TPyP	Mn^2+^	H-MnO_2_@TPyP@Bro	Hollow sphere, 240	MCF-7	MCF-7	20% (300)	Producing O_2_, Bro promotes the accumulation of H-MnO_2_@TPyP@Bro	77, 2022
4-DCF-MPYM	Cu^2+^	CaO_2_-FM@Cu-ONS@HC	Sphere, 120.4	HeLa, 4T1, COS-7	4T1	10% (100)	O_2_/H_2_O_2_ self-sufficient	78, 2022
								
Fe/Mn-ZIF-8	Fe^2+^/Mn^2^	lanthanide-doped NPs @Fe/Mn-ZIF-8	Sphere, 75.4	HeLa	U14	10.2% (500)	Dual doping of Fe^2+^/Mn^2+^ decreases the bandgap of the ZIF-8 photosensitizer/ GSH depletion	84, 2022
**CDT-PTT**								
Carbon dots	Fe^2+^/ Fe^3+^	RCDs@MIL-100	Polyhedral, 180	4T1, HC11	4T1	5% (1000)	GSH depletion/ hyperthermal-enhanced CDT	89, 2022
								
BODIPY	Fe^2+^	(BODIPY)-Fe (III)	Spherical, 40	HeLa	HeLa	10% (50)	Outstanding Fenton catalytic performance/ strong NIR-II absorbing ability	91, 2020
								
CuS	Cu^2+^	Cu-MOF [Cu- (bpy)_2_(OTf)_2_]	Square, side lengths 1.2 μm	CT-26	CT-26	19.1% (500 uM)	H_2_S consumption, CuS generated in situ, smaller CuS enhances Fenton-like reaction	96, 2022
								
CoS QDs	CoS QDs	CoS QDs	5.8 nm	LO2, A431, MDA-MB-231, 4T1	4T1	4T1:20% (1 mM) A431:40% (1 mM)	Regulating the photothermal conversion efficiency, promoting the Fenton catalytic capability, hyperthermal-enhanced CDT	100, 2022
**CDT-SDT**								
Fe-porphyrin	Fe^2+^	MMSN@Au-Fe(TPP)@LM	Spiky, 302.2 ± 1.4	HUVEC, B16F10	B16F10	20% (200)	Producing H_2_O_2_, US augmentes cascade-catalytic	112, 2022
								
Ce6	Cu^2+^	Cu/CaCO_3_@Ce6	Approximately spherical	4T1	4T1	<10% (100)	self-supply of oxygen, Ca^2+^ overloading-sensitizes CDT/SDT, GSH deprivation	114, 2022
								
TiO_2_/Ti_3_C_2_	Cu^2+^	Ti_3_C_2_/CuO_2_/BSA	Nanosheet,189	U87	U87	<40% (Ti:50 μg/mL)	In situ generation of sonosensitizers, H_2_O_2_ generation, enhanced separation of e-and h+	118, 2022
								
BaTiO_3_	Cu^2+^	Cu_2-x_O-BTO	Cubic, 162.3 ± 3.5	NIH-3T3, 4T1	4T1	18.9% (400)	Continuous accumulation of electrons and holes, electron-hole pairs separation and migration	119, 2022
								
Au NPs	Mn^2+^	Au-MnO NPs	Snowman, 20	MCF-7	97H	<40% (200)	Generation smaller Au NPs in situ, numerous cavitation nucleation sites	121, 2020
**CDT-RT**								
CaO_2_/Fe^3+^/ ZIF-8)	Fe^2+^/ Fe^3+^	CaO_2_/Fe^3+^/ ZIF-8	Spherical, 45.52	patient-derived cancer cells	patient-derived cancer cells	<25% (200)	Self- sufficient H_2_O_2_, O_2_	125, 2020
Hf-BPY	Fe^2+^	(Hf-BPY-Fe)	Octahedral, 100	HeLa	HeLa	24.5% (80 ppm)	Electron-rich environment accelerates the reduction from Fe^3+^ to Fe^2+^	131, 2020
								
SnS2/Fe_3_O_4_	Fe^2+^/ Fe^3+^	SnS2@Fe_3_O_4_ NPs	Hetero-geneous nanoparticle	HeLa, HUVECs	HeLa	40% (90)	X-ray enhances Fe^2+^/Fe^3+^ cycling for CDT	134, 2021
								
SPIONCs	Fe^2+^	SPIONCs	Spherical-like, 60-200	NCI-H460	H460	28.9% (90)	Increasing the production of H_2_O_2_	136, 2022
**CDT-MHT**								
iron oxide	Fe^2+^	GOD/iron oxide nanocatalysts	flower-like	PC3	PC3	<10% (100)	Down-regulate HSP expression, supplying abundant H_2_O_2_	148, 2020
								
Fe_3_O_4_	Fe^2+^	Fe_3_O_4_	Nanospheres, 300	4T1	4T1	<40% (100)	Suppressing the expression of HSP70 and HSP90, heat facilitates CDT	149, 2020
								
MnFe_2_O_4_	Fe^2+^	Ir@ MnFe_2_O_4_	NPs: 11.24 ± 1.11 nm	HeLa	HeLa	14% (400)	depletion of GSH, Enhanced cell sensitivity to MHT	150, 2020
CDT-immunotherapy								
αPDL1	Fe^2+^/ Fe^3+^	GOx/αPDL1/OEGCG/Fe^3+^/POEGMA-b-PTKDOPA	110.3 ± 7.2	4T1	4T1	10% (GOx: 1 U/mL)	Producing H_2_O_2_, enhanced immunogenic cell death	161,2022
								
Bacterium substrate	Au@Pt	E. coli/Au@Pt	core-shell,575	HeLa, COS-7, HepG2, B16-F10	B16-F10	IC50 :0.6 ppm	Weaken the GSH, tumor targeting ability of bacteria	164, 2021
								
Ferrocene - MOF/Vk3	Fe^2+^	Ferrocene - MOF/Vk3	150.2 ± 22.6	L929, 4T1	4T1	15% (Vk3:100)	Vk3- mediated H_2_O_2_ producing, promoting DC maturation	165, 2023
								
MnOx	Mn^2+^	MnOx- ovalbumin	Nanospikes	4T1	4T1	30% (800)	Ultrahigh loading efficiencies for ovalbumin and tumor cell fragment	168, 2020
**CDT-ST**								
GOD	Mn^2+^	Mn_3_O_4_/GOD co-loaded organosilicon	Spherical, 50	3T3, SMMC-7721	SMMC-7721	12.7% (75)	supplying O2 and H_2_O_2_	174, 2023
								
Au NPs	ZIF-67 (Co^2+^)	ZnO2@Au@ZIF-67	Spherical, 60	4T1, NIH/3T3, HUVEC	4T1	<10% (4)	generating O2 and H_2_O_2_	175, 2023
								
LOX, TA-Fe (III)	Fe^2+^	PFOB/ LOX-TA- Fe (III)	core-shell,182 ± 13	4T1, CT26, MCF-10A	4T1	20% (LOX: 1.2)	dual-depletion of lactate and ATP, O2 and H_2_O_2_ self-supply	181, 2021
**CDT-GS**								
NO	Cu^2+^, Mo^4+^	Mo/Cu9S5/ L-Arginine	Spherical with huge cavity	4T1, L929	4T1	30% (200)	Depletion of GSH, ultrasound enhances NO release, NO inhibits protective autophagy	184, 2022
								
CO	Mn^2+^	MnCo@UiO-67-bpy@GOx	Spherical, 90	HeLa, L929 MCF- 7	HeLa	7% (160)	Producing H_2_O_2_, accelerating CO release	185, 2022
								
H_2_S	Fe^2+^	FeS@BSA	Spherical, 50	Huh7, WRL-68	Huh7	20% (20)	H_2_S induced H_2_O_2_ amplification	186, 2020
**CDT-GT**								
siS100A4	Mn^2+^	ErNPs@MnO2-siS100A4-RGD	Spherical, 50	MDA-MB-231, MCF-10A	MDA-MB-231	10% (20)	GSH-depletion, superior tumor-targeting	197, 2021
								
DNAzyme	Cu^2+^	DNAzyme-Cu^2+^-TA	Spherical, 200	4T1	4T1	25% (200)	depletion of GSH, ultra-high loading capacity	205, 2021
**CDT-OT**								
CaCO_3_	Fe^2+^	calcium-and iron-doped silica loaded with DHA	Spherical, 80	MDA-MB-231, 4T1	4T1	10% (200)	Fe^2+^ interacted with DHA to generate C-centered radicals to amplify CDT	209, 2022
**CDT-based other combinations**								
ITT	Cu^2+^	CaO_2_-CuO2@HA	Spherical,120	4T1, CT26, B16F10, L929	4T1	17.5% (120)	H_2_O_2_ self-supplying, GSH depletion	212, 2022
								
EDT	W^5+^	POM@ZIF-8	Spherical, 210	HeLa	HeLa	30% (100)	Enhanced ROS levels	214, 2022
**CDT in combination with trimodal therapy**								
CDT- chemotherapy-PTT	Fe^2+^	Mitoxantrone- -GOx@*γ*-Fe_2_O_3_	Spherical, 86.2	4T1	4T1	10% (11.52)	H_2_O_2_ amplification	216, 2022
								
CDT-ST- chemotherapy	Mn^2+^	zeolitic-imidazolate- framework@MnO_2_/Dox	Polygonal,230	HeLa, HUVEC	HeLa	30% (200)	Reducing the recombination rate of e^-^ and h^+^, Producing O_2_	217, 2022
								

## Abbreviations

CDT: chemodynamic therapy
TME: tumor microenvironment
ROS: reactive oxygen species
DDS: drug delivery system
MOF: metal-organic framework
ASA: artesunate
MTX: methotrexate
PD-L1: programmed death ligand-1
CTLA-4: cytotoxic T-lymphocyte associated protein 4
TAAs: tumor associated antigens
ICD: immunogenic cell death
NOx: nicotinamide adenine dinucleotide phosphate oxidases
SOD_2_: manganese superoxide dismutase
PDT: photodynamic therapy
NPs: nanoparticles
PTT: photothermal therapy
RT: radiation therapy
SDT: sonodynamic therapy
MHT: magnetic hyperthermia therapy
OT: oncosis therapy
HSPs: heat shock proteins
siRNA: small interfering RNA
